# Target-Driven Evolution of Scorpion Toxins

**DOI:** 10.1038/srep14973

**Published:** 2015-10-07

**Authors:** Shangfei Zhang, Bin Gao, Shunyi Zhu

**Affiliations:** 1 Group of Peptide Biology and Evolution, State Key Laboratory of Integrated Management of Pest Insects & Rodents, Institute of Zoology, Chinese Academy of Sciences, 1 Beichen West Road, Chaoyang District, 100101 Beijing, China

## Abstract

It is long known that peptide neurotoxins derived from a diversity of venomous animals evolve by positive selection following gene duplication, yet a force that drives their adaptive evolution remains a mystery. By using maximum-likelihood models of codon substitution, we analyzed molecular adaptation in scorpion sodium channel toxins from a specific species and found ten positively selected sites, six of which are located at the core-domain of scorpion α-toxins, a region known to interact with two adjacent loops in the voltage-sensor domain (DIV) of sodium channels, as validated by our newly constructed computational model of toxin-channel complex. Despite the lack of positive selection signals in these two loops, they accumulated extensive sequence variations by relaxed purifying selection in prey and predators of scorpions. The evolutionary variability in the toxin-bound regions of sodium channels indicates that accelerated substitutions in the multigene family of scorpion toxins is a consequence of dealing with the target diversity. This work presents an example of atypical co-evolution between animal toxins and their molecular targets, in which toxins suffered from more prominent selective pressure from the channels of their competitors. Our discovery helps explain the evolutionary rationality of gene duplication of toxins in a specific venomous species.

Voltage-gated sodium (Na_v_) channels are transmembrane protein complexes mainly comprising one pore-forming α-subunit and one or two smaller auxiliary β-subunits (β1–β4) that modulate the kinetics and voltage dependence of channel gating. The α-subunit constitutes an essential functional unit of the channel and usually contains more than 2,000 amino acids with four highly homologous but not identical structural domains (DI to DIV), each of which includes six α-helical segments (S1–S6) that are long enough to cross the membrane and a reentrant pore loop (P) between S5 and S6^1^,^2^,^3^,^4^. The first four-helix bundle (S1–S4) forms a modular voltage-sensor domain (VSD) to initiate channel activation, and S5, S6 and the intervening P-loop form the pore domain allowing Na^+^ to across the membrane. Na_v_ channels are responsible for the generation and propagation of action potentials in nerves, muscles, and other excitable cells^5^. Abnormal Na_v_ channel action has been linked to various neurological and cardiac disorders^6^,^7^.

Given key physiological roles in the excitability of neurons and muscles, Na_v_ channels have been evolutionarily selected as targets by many venomous animals. Toxins from venoms bind to at least nine different receptor sites in the α-subunit, in which sites 3 and 4 are primarily targeted by scorpion α- and β-toxins^8^,^9^. These toxins contain 61–76 residues with four disulfide bridges and they fold into a typical cysteine-stabilized α-helix and β-sheet (CSαβ) scaffold consisting of one α-helix and one triple-stranded β-sheet^10^. Scorpion α-toxins are the firstly identified venom component with lethal effect on both insects and mammals. They are only present in species of the Buthidae family^11^ and cause a slowing of the inactivation of Na_v_ channels by binding to site 3^1^,^12^. According to their preference for either insect or mammalian Na_v_ channels, α-toxins can be further divided into three distinct subgroups: the classical α-subgroup, highly active on mammalian brain Na_v_ channels; the insect α-subgroup, highly specific for insects; and the α-like subgroup, toxic on both mammalian and insect Na_v_ channels^11^,^12^.

The adaptive evolution of the scorpion α-toxin family has been an intriguing model for studying functional diversification of proteins via accelerated substitutions in the bioactive surface^13^,^14^. The pioneering analysis of this family by maximum likelihood (ML) models of codon substitution identified 11 sites evolved by positive selection^13^. Subsequently, a similar analysis was reported based on sequences from more scorpion species^15^. To investigate the evolutionary significance of positive selection of toxins in a specific lineage, Zhu *et al.* re-analyzed α-toxins from two sibling scorpion species (*M. eupeus* and *M. martensii*) and detected nine positively selected sites (PSSs) that are associated with functional diversification of *Mesobuthus* α-toxins^14^. More recently, Sunagar *et al.* showed that α-toxins from several Australian scorpion species had experienced episodic influence of positive selection with 14 sites evolved by positive selection^16^. Despite these remarkable progresses, it is still unclear what factors have driven the adaptive evolution of these toxins in a specific species.

Here, we report the discovery of PSSs in scorpion α-toxins from a single species (*Mesobuthus martensii*) and their locations in the toxin-channel interface. In combination with structural and evolutionary analyses of Na_v_ channels from both prey and predators of scorpions, we provide convincing evidence for an atypical co-evolutionary manner between scorpions and their competitors, in which toxin-bound regions of the ion channel evolved by relaxed purifying selection to accumulate sequence mutations may act as a driver of the adaptive evolution of toxins. Our work therefore addresses a key question regarding the evolutionary rationality of the presence of multiple paralogous α-toxins in a scorpion’s venom.

## Results and Discussion

### Positively Selected Sites of M. martensii Sodium Channel Toxins

**Figure 1:**
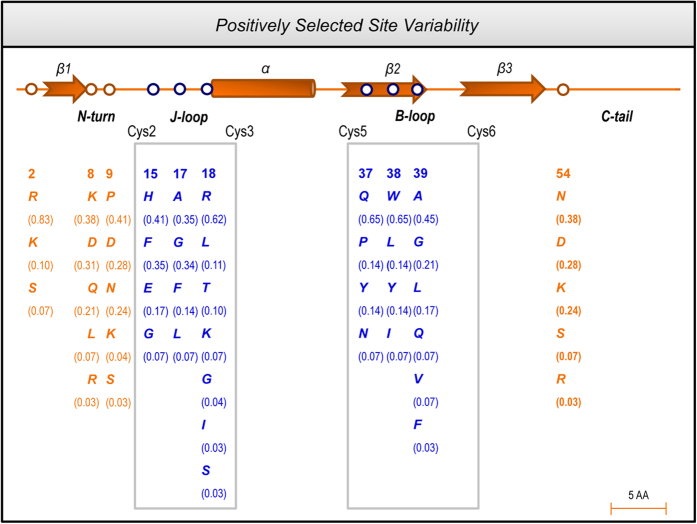
Mapping of PSSs of the *M. martensii* α-toxins on the secondary structure of BmKM1. Residue variability of PSSs is shown in *blue* for the core-domain and *orange* for the NC-domain. Relative frequencies of each PSS are shown in bracket. Only the PSSs convergently predicted by M2a and M8 are shown here. The core-domain refers to two loops (boxed in *grey*): J-loop, the region between Cys2 and Cys3, structurally preceding the α-helix; B-loop, the region between Cys5 and Cys6, structurally linking two β-strands. The NC-domain includes the N-turn and C-tail.

The completion of the whole genome sequencing of the first scorpion species (*M. martensii*)^17^ allows us to gather nearly all scorpion α-toxin genes of this species to analyze their evolutionary selection pattern and potential driving force. A total of 29 non-redundant toxin sequences were collected and aligned, which cover all three different pharmacological subgroups, e.g. α-like BmKM1 and BmKM10, insect-specific BmKαIT1, and classic BmKαTX11 (Fig. S1). To test positive selection of the *M. martensii* α-toxin multigene family, we employed maximum likelihood (ML) models of codon substitution to identify potential PSSs (sites with a nonsynonymous to synonymous substitution rate ratio, *d*_*N*_/*d*_*S*_ = ω, > 1 significantly)^18^. As shown in Table 1, the ML estimates (MLEs) under M0 predicts that all amino acid sites have a ω of 0.85, approximately equals 1, indicative of neutral selection. However, M0 fits the data worse than other models, suggesting that ω values (i.e. selective pressure) may be heterogeneous among sites. The MLEs under M2a suggest that 27% of sites are under positive selection with ω = 2.47 (Table 1). The likelihood ratio test (LRT) statistic between M1a and M2a is 16.6 (2Δl), much greater than critical values from a χ^2^ distribution with degree of freedom = 2 (*p* < 0.001), indicating the presence of PSSs. M2a did identify 10 PSSs with P ≥ 0.9 by methods of the Bayes Empirical Bayes (BEB) (Table 1) and the Naïve Empirical Bayes (NEB) (Table S1). The test using M7 and M8 models results in a highly similar conclusion. Although M8 predicts two more PSSs, 10 of them are identical to those predicted by M2a (Table 1 and Table S1). It is remarkable that these PSSs are scattered in the primary structure of the toxin, yet they cluster in the functional regions previously characterized^19^,^20^,^21^,^22^, in which six (15E, 17A, 18R, 37Q, 38W and 39V, numbered according to BmKM1) are seated on the core-domain comprising two loops: B- and J-loop; and others on the NC-domain (Fig. 1). When compared with the PSSs identified previously^13^,^14^,^15^, it was found that the majority of them were overall similar in positions, suggesting that Na_v_ channel toxins from different scorpion species might suffer from similar selective pressure.

Analysis of amino acid composition of the PSSs revealed their high variability (Fig. 1), as identified by nearly all PSSs containing different polar and non-polar amino acids and some even are occupied by residues with opposite charges. For example, at site 15 there are four different types of amino acids (Glu, Gly, His and Phe), varying from small neural glycine to large aromatic phenylalanine, and from positively charged histidine to negatively charged glutamic acid.

### PSSs Located at the Toxin-Channel Interface

**Figure 2:**
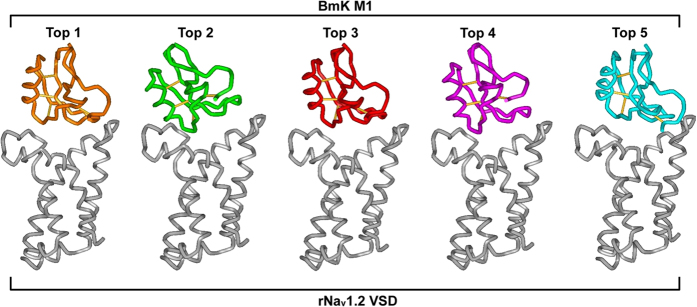
Five top ZDOCK complexes between BmKM1 and the rNa_v_1.2 VSD. These complexes are displayed in a tube style with WebLabViewer (MSI, San Diego, California, USA). Disulfide bond connectivities in BmKM1 (pdb entry 1SN1) are shown as sticks. The toxin has only 3.77 Å of root-mean-square deviation (RMSD) among different complexes, as calculated by Swiss-PdbViewer (http://spdbv.vital-it.ch/).

Previous experiments have indicated that two extracellular loops linking S1–S2 and S3–S4 in the domain IV VSD of Na_v_ channels (L_DIVS1-S2_; L_DIVS3-S4_) are main regions of site 3 directly interacting with the core-domain of α-toxins^23^,^24^. The pore module of domain I in Na_v_ channels forms a secondary site by the interaction with the NC-domain of toxins^22^,^24^,^25^,^26^,^27^. To observe the location of the PSSs in the interface of toxin-channel, we constructed a complex model between BmKM1, an extensively studied and structurally known *M. martensii* α-like toxin, and the DIV VSD of rNa_v_1.2 using the ZDOCK server^28^. The channel structure used here is the one previously successfully used in exploring the binding mode of a scorpion α-toxin (AaHII)^29^. As shown in Fig. 2, in the five top ZDOCK models, BmKM1 similarly binds to the VSD (Fig. 2) and we thus selected the top 1 model for further analysis.

**Figure 3:**
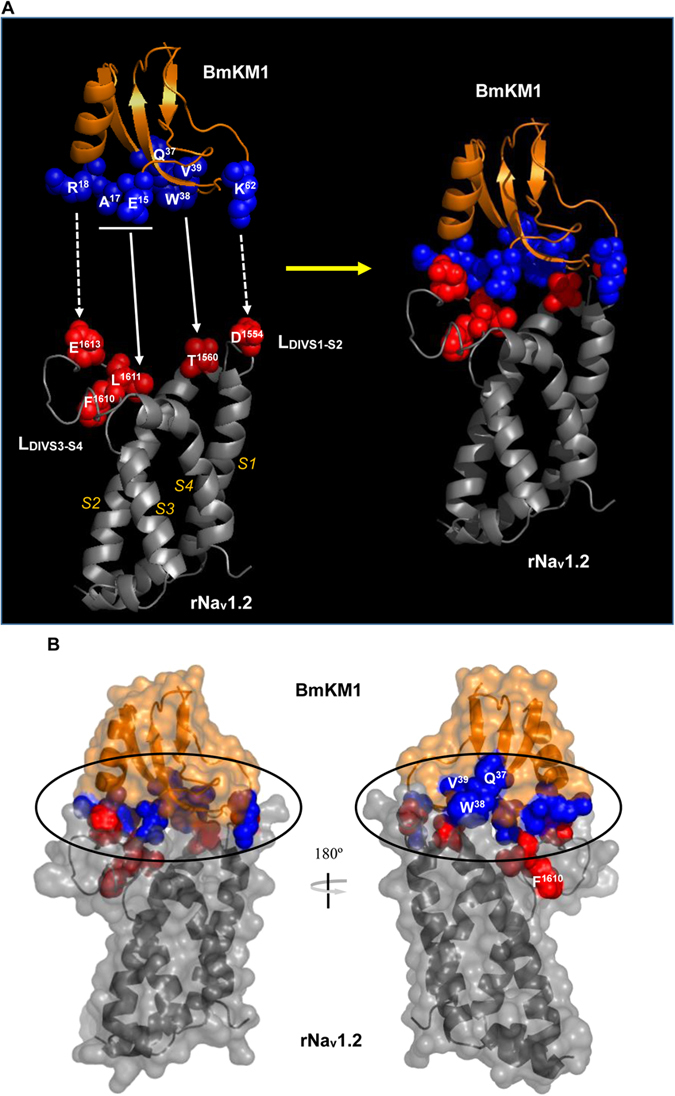
Detailed structural analysis of top 1 model of the ZDOCK complexes. (**A**) Ribbon models showing interactions between PSSs of toxins and residues derived from the VSD of rNa_v_1.2, previously identified as a primary component of the receptor site for α-toxins (spheres in *blue*, PSSs and *red*, channel residues). Predicted contact residues between the toxin and the channel are indicated by arrows and dotted arrows illustrate residues forming salt bridges; (**B**). Molecular surface of BmKM1 and the VSD of rNa_v_1.2 showing their interface (cycled), in which a ribbon structure of the backbone with side-chain atoms (space-filled) of PSSs and the channel residues is covered by a semi-transparent surface of the complex. The orientation of the ribbon structure is the same with that of Fig. 3A.

In the complex structure, four toxin-VSD residues pairs were identified to contact directly, including: *1*) Glu15/Ala17-L1611; *2*) Arg18-Glu1613; *3*) Trp38-Thr1560; and *4*) Lys62-D1554, where pairs 2 and 4 form salt bridges to stabilize the complex structure (Fig. 3A). Evidences supporting the reliability of this complex come from the following observations: *1*) Functional importance of the toxin residues (i.e. sites 15, 17, 18, 38 and 62) in the interface of the complex has been verified by prior mutagenesis data in multiple toxins, such as BmKM1, Lqh2, Lqh3 and LqhαIT^19^,^20^,^21^,^22^,^23^,^24^,^30^; 2) Thr1560 and Glu1613 have been found to be key residues of rNa_v_1.2 implicated in Lqh2 binding and Glu1613 was previously identified as a primary component of the receptor site for α-toxins^24^,^25^,^31^; *3*) Pairs 1, 2 and 4 were also observed in the AaHII-rNav1.2 VSD model that was built based on atomistic molecular dynamics simulations^29^; *4*) The overall orientation of the toxin relative to the VSD in our complex is similar to models of Lqh2-rNav1.2^25^ and AaHII-rNav1.2. In these three complexes, residues from the toxin’s J-loop are close to L_DIVS3-S4_ of the channel and residues from the toxin’s B loop to L_DIVS1-S2_ (Fig. 3A). Apart from the four residues mentioned above implicated in direct contact with the channel, two additional PSSs (Q37 and V39) are also located on the interface of the complex (Fig. 3B). Only one exception in our complex is F1610, a channel residue derived from L_DIVS3-S4_ of rNa_v_1.2 conferring to toxin binding^25^, is a bit far away from the interface (Fig. 3B). A possible explanation is that this residue directly interacts with the toxin through a conformational adjustment, as proposed in the Lqh2-rNa_v_1.2 model^25^. Alternatively, this residue might have a synergistic role with its adjacent residue (e.g. L1611) to bind to the toxin (Fig. 3B).

### Relaxed Purifying Selection in Toxin-Bound Regions

As a class of small-sized venomous animals, scorpions exist at an intermediate level in food chains. They prey on a variety of small arthropods (insects and spiders) as their foods; in the meantime, they are prey to a diversity of larger vertebrate predators, such as birds (37 percent of all vertebrate predators), lizards (34 percent) and mammals (18 percent)^32^. To investigate whether these prey and predators have driven positive selection of scorpion α-toxins, we analyzed the evolutionary pattern of their Na_v_ channels in the toxin-bound region, which include 84 members from birds, 28 from lizards, 27 from mammals and 45 from insects (Fig. S2–Fig. S5).

**Figure 4:**
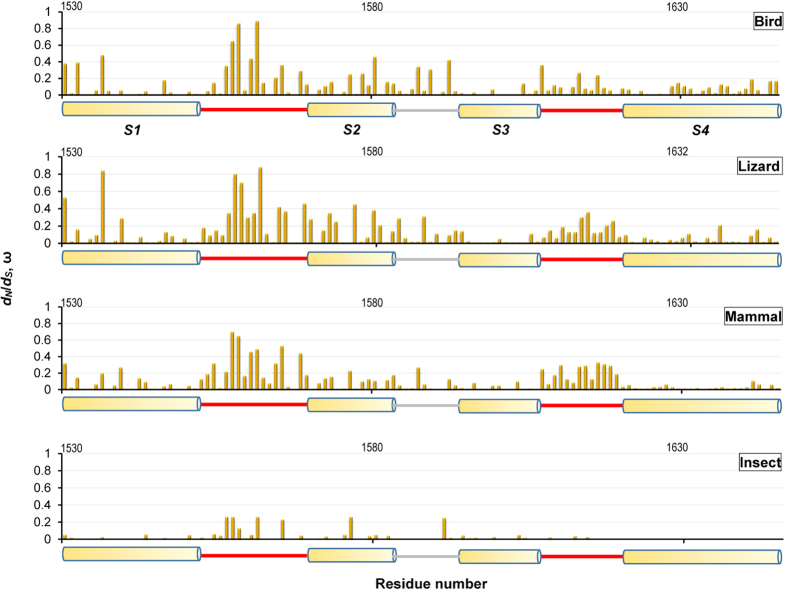
Divergence rates (ω) for each site of VSD from both predators and prey of scorpions. The ω values are calculated on Selecton under M8a model. For clarity, all sequences are numbered according to rNa_v_1.2. Secondary structural elements are extracted from the rNa_v_1.2 VSD model^29^ where α-helices are shown as cylinder, two extracellular loops involved in toxin binding as lines in *red*, and the intracellular loop in *grey*.

Firstly, we used the same ML models of codon substitution to test selective pressure in the VSD from the four different lineages (birds, lizards, mammals and insects) and, unexpectedly, we detected no any signals for their adaptive sequence evolution. The MLEs under M0 suggest that average ω ratios for overall sequence pairs in these lineage ranged from 0.02 to 0.09, far smaller than 0.85 in the toxin gene (Tables 1, 2, 3, 4, 5), indicating strong purifying selection on the VSD, consistent with the prediction of M2a and M8 that detected no sites evolved by positive selection. Although M8 fits the data better than M7 in the bird Na_v_ channel (2Δl = 11.6, 0.002 < *p* < 0.005), the extra class of sites (3%) has a ω_s_ = 1 rather than >1 (Table 2). In the mammalian and insect Na_v_ channels, the MLEs under M8 gave ω_s_ > 1, but their proportion (*p*1) equals 0 (Tables 4 and 5). Hence, no site was positively selected in these genes. Comparison of the log likelihood values revealed that M0 fits the data worse than other models in all the lineages (Tables 2, 3, 4, 5), supporting the presence of variable selective pressure among sites of the VSD. We therefore calculated ω values for each site in the VSD of different lineages by Selecton, a server for detecting evolutionary forces at a single amino-acid site, under M8a^33^. This model is a variation of M8 with the ω_s_ of the extra class sites set to 1 other than >1 in M8^18^,^34^ and thus suitable for testing purifying and neutral selection in each site (0 < ω ≤ 1). As shown in Fig. 4, each site has different evolutionary rates and in particular a significantly higher ω was observed in sites belonging to the two extracellular loops (L_DIVS1-S2_ and L_DIVS3-S4_) than those in their respective adjacent helical segments in the predators (Fig. 4). In insects, a similar case was also observed in L_DIVS1-S2_. The elevated evolutionary rates in the loops suggest they are under lower selective constraints than the helices, evidence for relaxed purifying selection. This could be ascribed to the distinct physiological role of VSD (DIV) in the Na_v_ channel fast inactivation, in which the helices are structurally and functionally important elements^35^ and they thus are expected to be constrained by stronger purifying selection to maintain their sequence and structural conservation. On the contrary, the two loops can tolerate more amino-acid substitutions in the absence of functional constraint.

**Figure 5:**
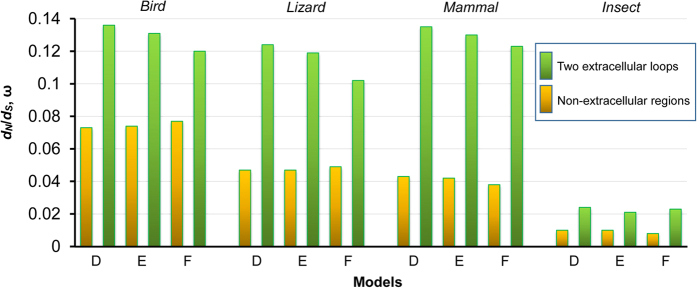
Different divergence rates between scorpion toxin-bound regions and non-bound regions of Na_v_ channels. Three fixed-sites models (**D**–**F**) were used to calculate ω values for two partitions of the VSD in Na_v_ channels from birds, lizards, mammals and insects: one partition representing the scorpion toxin-bound regions (two extracellular loops, bars in *light green*) and the other the four transmembrane helices and the intracellular loop (bars in *orange*).

To provide further evidence for relaxed purifying selection in the toxin-bound regions (L_DIVS1-S2_ and L_DIVS3-S4_) of Na_v_ channels, we employed six fixed-sites models (A to F, from simple to complex) developed by Yang and Swanson^36^ to compare their ω values with those of the non-toxin-bound regions (S1–S4 and the intracellular loop) from the four different lineages. As shown in Supplementary Tables 2 to 5, in all the lineages, the three more complex models (D, E, and F) that assume different ω ratios between two partitions convergently fit the data better than the three more simple models (A, B, and C). For example, the LRT statistics between C and E are 14.4 for birds, 33.0 for lizards, 43.2 for mammals, and 12.4 for insects (p < 0.005) (Table S2–Table S5). These models all suggest that the two partitions have very different ω values and overall the toxin-bound regions evolved two folds more quickly than its neighboring regions (Fig. 5), in line with the opinion of relaxation of purifying selection in these extracellular loops (Fig. 4).

### Amino Acid Variability in Toxin-Bound Regions

**Figure 6:**
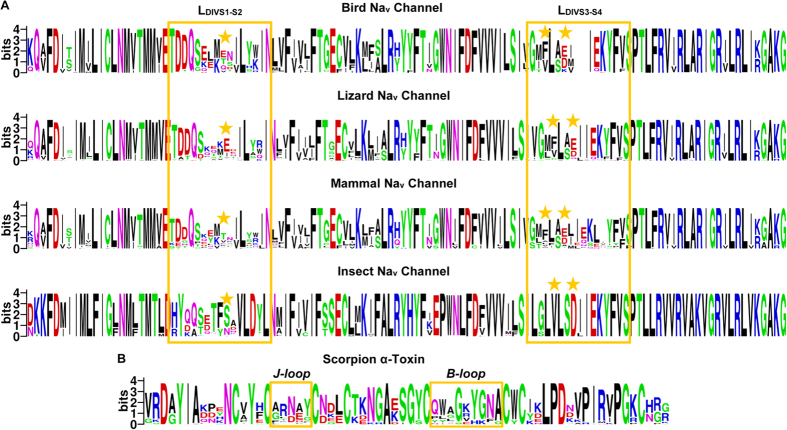
Sequence logos. (**A**) Na_v_ channel VSDs from birds, lizards, mammals and insects; (**B**) Scorpion α-toxins. Each logo consists of stacks of letters and the overall height of each stack indicates the sequence conservation at that position (measured in bits). The height of symbols within the stack reflects the relative frequency of the corresponding amino acid at that position^39^. Loops involved in toxin-channel interaction are boxed in *orange* in both toxins and channels; and positions of amino acids implicated in binding of rNa_v_1.2 to Lqh2^25^ are labeled by asterisks.

**Figure 7:**
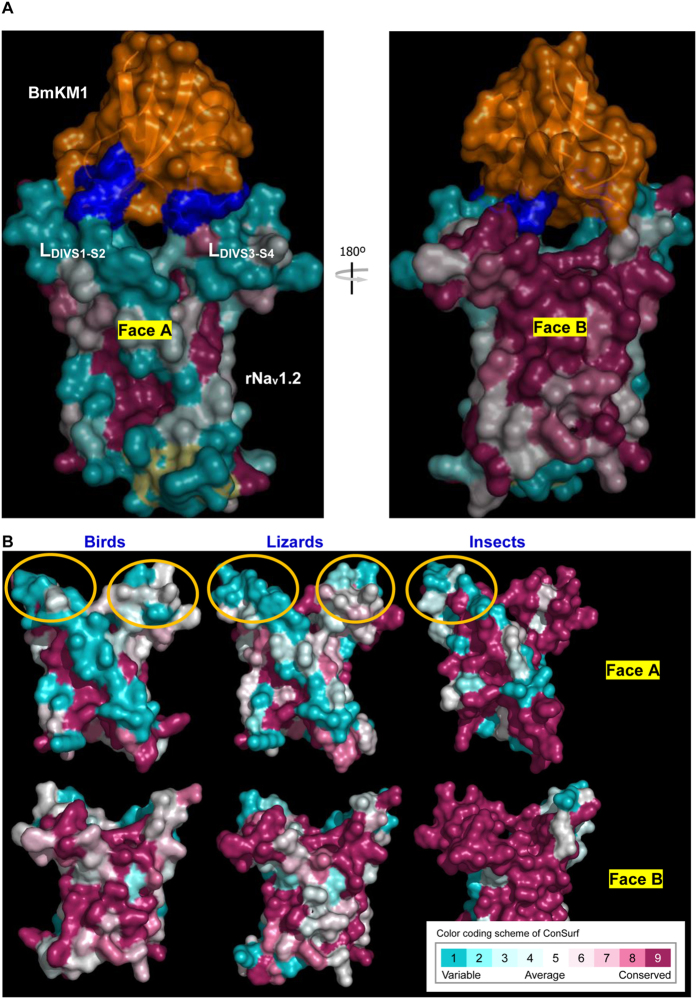
High variability of scorpion α-toxin-bound regions in Na_v_ channels from both predators and prey of scorpions. (**A**) Molecular surface display shows that BmKM1 binds to the evolutionarily variable loops (L_DIVS1-S2_ and L_DIVS3-S4_) of the VSD via its PSSs (*blue*). The complex model is the same with that of Fig. 3; (**B**) Molecular surfaces of VSDs from birds, lizards and insects. Variable regions involved in toxin binding are circled. Consurf (http://consurf.tau.ac.il/) was introduced to compute the position-specific conservation scores in the VSD and colored according to the scores. Structures of bird, lizard and insect VSDs are modeled from sequences of *Picoides pubescens* (gi|699624821), *Anolis carolinensis* (gi|343098400) and *Drosophila melanogaster* (gi|403447) (Figs. S2, S3 and S5) based on the previously reported rNa_v_1.2 model^29^. Faces A and B are two faces of the VSD rotated 180° around y axis.

Given the extensive existence of co-evolution between proteins and their interaction partners (e.g. ligand-receptor pairs)^37^,^38^, the lack of matched PSSs in the toxin-bound region of Na_v_ channels is puzzling as strong positive selection signals exist in the channel-bound region of the toxins (Fig. 3). To address this puzzling, we further analyzed sequence conservation in the VSD using WebLogo, a web-based application designed to make the generation of sequence logos^39^. The results indicate that the two loops (L_DIVS1-S2_; L_DIVS3-S4_) are highly variable in birds, lizards and mammals relative to their adjacent transmembrane helices (S1–S4) that show more conservation (Fig. 6A). In insects, more variability was also found in L_DIVS1-S2_. In parallel, we calculated the sequence logo of the toxin family, confirming the variability of the two positively selected loops (B- and J-loop) (Fig. 6B). The evolutionary variability of the toxin-bound region in VSDs of the Na_v_ channels is further confirmed by ConSurf, an algorithmic tool for the identification of variable and conserved regions in proteins by surface mapping of phylogenetic information^40^. As shown in Fig. 7A, BmKM1 binds to the two variable loops of the mammalian VSDs primarily via its PSSs (shown in *blue*). In the other two vertebrate predators (birds and lizards), the variability also occurs in similar regions of their VSDs. In accordance with the sequence logo, the insects have only one variable loop (Fig. 7B). These results suggest that the amino acid variability observed within the VSD in predators and prey of scorpions is a consequence of relaxed purifying selection, leading to their higher evolutionary rates.

### Channel’s Variability Driving Adaptive Evolution of Scorpion Toxins

**Figure 8:**
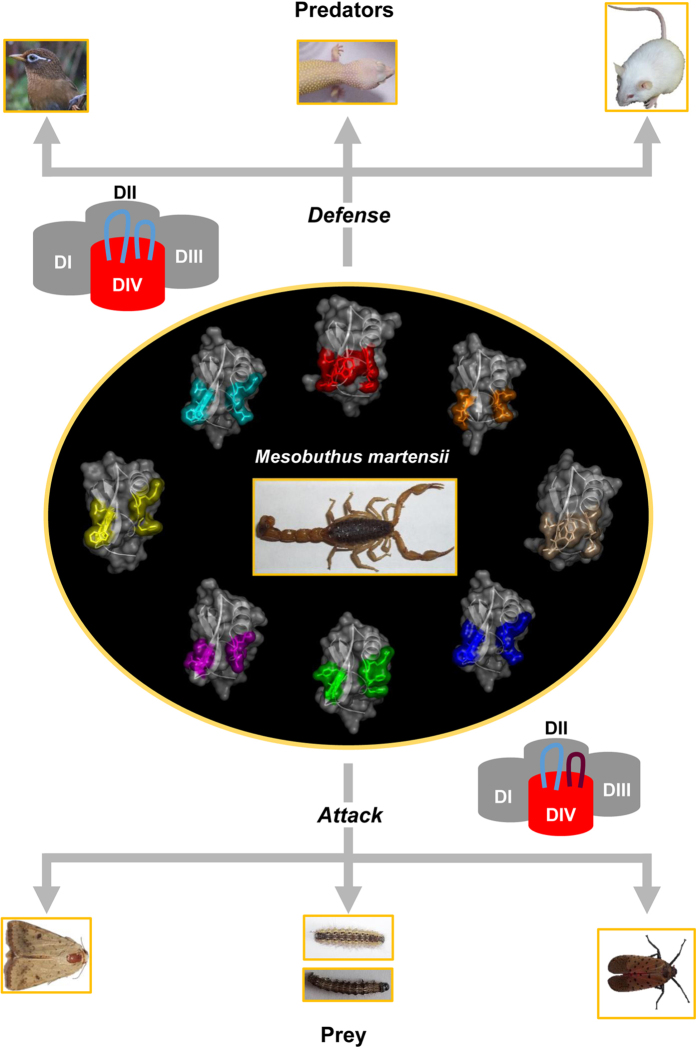
Predators and prey driving accelerated evolution of scorpion α-toxins. Birds, lizards and mammals are selected as representatives of scorpion’s predators and insects as a representative of scorpion’s prey. The toxins shown here all stem from *M. martensii* with the core-domain-derived PSSs in different colors, including *cyan* (BmKM1); *red* (BmKM4); *brown* (BmKM7); *tint* (BmKM8); *blue*, BmKαTX10; *green*, BmKαTX17; *pink*, BmKmX4; and *yellow*, BmKSCT (Fig. S1). Evolutionarily variable and conserved loops are colored *cyan* and *brown*, respectively, in the channel schematic diagram. The image of the bird was photographed by Prof. Sun Yuehua and others by the authors.

In the toxin-channel complex, we have established several pairs of interactions, in which three channel sites located in the two loops are involved in interaction with the PSSs of toxins, including 1560, 1611 and 1613 (Fig. 3), and two of them exhibit a side-chain variability: 1560: E/K/Q/T/V in birds, D/E/I/K/T/V in lizards, E/D/K/S/T/V in mammals, N/S/T in insects; 1613: E/D/G/K in birds, E/D/G/K/Q in lizards, A/D/E/G/T in mammals. Besides these point mutations, L_DIVS3-S4_ also contains some insertion/deletion (indel) mutations in birds and mammals (Figs. S2–S4). Collectively, these variable toxin-bound sites derived from the predators and prey may drive accelerated changes of their interacting residues (PSSs) in scorpion toxins for maintaining abilities in both defense and attack during evolution (Fig. 8). These observations also account for evolutionary rationality of gene duplication of scorpion toxins in a specific species since multiple toxin members may facilitate to deal with a diversity of competitor species via rapidly changing their bioactive surfaces by positive selection (Fig. 8). Given that the majority of animal toxins exist in a multigene form^41^, our finding may be of general significance in understanding forces driving the evolution of these toxins in diverse venomous species.

The conservation analyses presented here indicate that the predators may exert greater selective pressure on scorpions than their prey as the toxin-bound region of these predators’ sodium channels display higher variability than their insect counterparts via point mutations and indels. Higher evolutionary divergence rates of the VSD in these vertebrate predators could be also related to their gene numbers. In insects and other invertebrates, Na_v_ channels exist as single or very low copy genes^9^,^42^,^43^ and this could lower their divergence rates among orthologous genes. In contrast to the prey’s counterpart, Na_v_ channels in the predators have undergone gene expansion to form a multigene family with differential tissue distribution^9^,^42^,^43^. In mammals, Na_v_1.1–Na_v_1.3 are mainly expressed in the central nervous system (CNS); Na_v_1.4 in skeletal muscles; Na_v_1.5 in cardiac muscles; Na_v_1.6 in both central and peripheral nervous system; Na_v_1.7 in the peripheral nervous system; Na_v_1.8 and Na_v_1.9 in sensory neurons. Therefore, to cope with these diverse Na_v_ channels from different tissues of the large-sized predators, scorpions need to evolve more paralogous toxins with altered bioactive surfaces (Fig. 8). However, it was found that scorpions seldom use venom to prey on small-sized insects^32^, in line with the opinion that insects may exert smaller impact to scorpions during evolution.

The discovery that scorpion α-toxins bind to evolutionarily variable regions of their targets is of rather importance in that it suggests that mutations at these regions by relaxed purifying selection might be a key factor driving the adaptive evolution of scorpion α-toxins. The potential role of purifying selection in promoting the evolution of protein-protein interfaces from cytochrome c oxidase I was recently reported^44^.

## Conclusions

Rapid evolution driven by positive selection has been observed in a variety of gene families, such as viper phospholipase A2, spider HSP70, vertebrate antimicrobial cathelicidin, neurotoxins from scorpions and cone snails^13^,^45^,^46^,^47^,^48^. However, driving forces responsible for their evolution remain unsolved. Based on a combination of analyses of the evolution of both toxins and channels, we provide evidence in favor of the variability of toxin-bound region in Na_v_ channels from predators and prey of scorpions as a driver for accelerated evolution of toxin functional regions following gene duplication (Fig. 8).

It has been proposed that co-evolution between predators and prey might engender evolutionary forces to motivate rapid diversification of functional surfaces of toxins^49^. However, the discovery of relaxed purifying selection in the toxin-bound region of Na_v_ channel raises a possibility of non-classical co-evolution, in which toxins are more selected to evolve rapidly for deterring physically huge predators^50^,^51^. On the contrary, the channels of predators and prey appear to experience no selective pressure from scorpions, as identified by the lack of positive selective signal in their toxin-bound regions. In addition to scorpion toxins, toxins from other venomous animals also target the two evolutionarily variable loops of Na_v_ channels^52^. Our conclusion might thus be of general significance in understanding the evolutionary rationality of gene duplication in venomous animals.

## Methods

### Sequence Analysis

This study includes sequences of 29 α-toxins, all derived from *M. martensii*; 27 Na_v_ channels from three mammalian species (*Mus musculus*, *Rattus norvegicus* and *Homo sapiens*), each consisting of nine paralogous genes, 45 from insects of eight orders, 84 from birds, and 28 from lizards. Nucleotide sequences were aligned by Clustal X (1.83) and corresponding alignments were used to construct neighbor-joint (NJ) trees for maximum likelihood (ML) models of codon substitution.

Multiple sequence alignments of toxins and channels generated by Clustal X were used to create sequence logos, a graphical representation for depicting conserved and variable regions within a protein family, by WebLogo^39^. To calculate conservation scores for sites in the *M. martensii* α-toxins and the VSD of Na_v_ channels from different lineages (birds, lizards, mammals and insects), we used the ConSurf program (http://consurf.tau.ac.il/) to analyze their amino acid sequences under default parameters. Similar to WebLogo, ConSurf also identifies both conserved and variable regions of a protein family, but it depends on not only sequence alignment but also phylogenetic tree. A Bayesian tree was constructed with the JTT evolutionary substitution model for each data^53^. Also, Consurf can clearly define the variability and conservation of proteins on their molecular surfaces.

### Positive Selection Analysis

It is generally accepted that the nonsynonymous to synonymous substitution rate ratio (ω = *d*_*N*_/*d*_*S*_) > 1 significantly is key evidence of protein adaptive evolution. To make a reliable test of positive selection, we employed two pairs of maximum likelihood (ML) models (M1a/M2a and M7/M8) to measure selective pressure among sites of the *M. martensii* α-toxins and the Na_v_ channels from different evolutionary lineages, in which the two null models (M1a and M7) do not allow sites with ω > 1 whereas their alternative models (M2a and M8) assume the existence of such sites (ω > 1)^54^. As M1a and M7 are nested within their respective alternative models (M2a and M8) and have two more parameters, the χ^2^ distribution may be used for a likelihood ratio test (LRT) to compare the fit between the two competing models. The Naïve Empirical Bayes (NEB) and the Bays Empirical Bayes (BEB) analyses were then used to calculate the posterior probabilities of ω classes for each site. The BEB is an improvement of NEB as it accounts for sampling errors in the ML estimates of parameters in the model^18^. Sites with a high probability (≥90%) of coming from the class with ω >1 are likely to be under positive selection and those located in the core-domain of scorpion α-toxins were used for next analysis. The ω values for each sites of the VSD in different lineages were calculated on the Selecton server^33^ under the M8a model^34^ and ω values between different partitions of the VSD were compared by fixed-sites models implemented in PAML^36^. In these analyses, the two extracellular loops of VSD for toxin binding is considered as one part while the remaining region as another part. Six fixed-sites models were used here: Model A, totally identical parameters including branch lengths, transition/transversion rate ratio κ, ω, and the nine parameters for the codon frequencies; Model B, different codon frequencies but equal different κ, ω and codon frequencies between two partitions; Model C, different codon frequencies and branch lengths but equal different κ and ω between two partitions; Model E, different κ, ω, branch lengths and codon frequencies between two different partitions; and Model F, separate analysis which assumes these two partitions have independent substitution parameters. The likelihood ratio text can be used to determine whether or not κ and ω are identical between two partitions^36^.

### Molecular Docking

ZDOCK (version 3.0.2), a Fast Fourier transform (FFT)-based, rigid-body protein-protein docking program^28^ (http://zdock.umassmed.edu/), was employed to construct the complex model between BmKM1 and the rNa_v_1.2 VSD. ZDOCK searches all possible binding modes in the translational and rotational space between the two proteins and performs scoring calculations based on a combination of statistical potential of interface atomic contact energies (IFACE), shape complementarity and electrostatics^55^. The atom coordinates of BmKM1 (PDB entry 1SN1) and the previously published structure of the VSD of rNa_v_1.2 (residues 1520–1645)^29^ were used as inputs for ZDOCK calculations. On the basis of previous mutational data both from the toxins and the Na_v_ channels, we specified binding site residues for filtering output predictions, which include sites 15, 17, 18, 38, and 40–43 in scorpion α-toxins (BmKM1, Lqh3, LqhαIT, and Lqh2)^14^; and sites 1560, 1610, 1613, 1617–1620 in the VSD of Na_v_ channels (rNav1.2 and rNa_v_1.4)^25^,^56^. All these sites are associated with the toxin-channel interaction, as identified by their mutations significantly affecting the binding of at least one toxin or channel mentioned here.

In terms of structural displays, unless otherwise indicated, structural figures presented here were prepared by PyMOL (www.pymol.org).

## Additional Information

**How to cite this article**: Zhang, S. *et al*. Target-Driven Evolution of Scorpion Toxins. *Sci. Rep.* **5**, 14973; doi: 10.1038/srep14973 (2015).

## Supplementary Material

Supplementary Information

## Figures and Tables

**Table 1:** Parameter estimates and likelihood ratio statistics (2Δ*l*) for the *M. martensii* α-toxin gene.

Models	*p*	*l*	Parameter estimates	2Δ*l*	PSSs
M0 (one-ratio)	1	−1497.0	ω = 0.85		None
M1a (neutral)	2	−1448.0	*p*_0_ = 0.46 (*p*_1_ = 0.54)		Not allowed
			ω_0_ = 0.08 (ω_1_ = 1)	16.6	
M2a (selection)	4	−1439.7	*p*_0_ = 0.42		2R, 8K^*^, 9P^*^, 15E, 17A,
			*p*_1_ = 0.31 (*p*_2_ = 0.27)		18R^**^, 37Q, 38W, 39V^**^,
			ω_0_ = 0.09 (ω_1_ = 1)		54N
			ω_2_ = **2.47**		
M7 (beta)	2	−1450.2	*p* = 0.20, *q *= 0.15	20.0	Not allowed
M8 (beta & ω)	4	−1440.2	*p*_0_ = 0.64 (*p*_1_ = 0.36)		2R^*^, 8K^**^, 9P^**^, 10H, 13V,
			*p* = 0.32, *q* = 0.58		15E^*^, 17A^*^, 18R^**^, 37Q^*^,
			ω_s_ = **2.21**		38W^*^, 39V^**^, 54N^*^

Note: *p* is the number of parameters in the ω distribution; *l* is the log likelihood. Twice the log likelihood differences (2Δ*l*) between null models and their alternative models: M1a/M2a = 16.6, M7/M8 = 20.0, both having χ^2^ significant values (p < 0.001). PSSs identified by the Bayes Empirical Bayes (BEB) methods under M2a and M8 with P (posterior probabilities) ≥ 0.9 are shown and those ≥ 0.99 are indicated by ^**^ and ≥ 0.95 by ^*^. The Naïve Empirical Bayes (NEB) methods gave similar results (Table S1). Residues are numbered according to BmKM1. Two ω values in M2a and M8, as indicators of positive selection, are boldfaced. M8-specific PSSs are underlined once.

**Table 2:** Parameter estimates and likelihood ratio statistics (2Δ*l*) for bird sodium channel genes.

Models	*p*	*l*	Parameter estimates	2Δ*l*	PSSs
M0 (one-ratio)	1	−6494.1	ω = 0.09		None
M1a (neutral)	2	−6407.5	*p*_0_ = 0.89 (*p*_1_ = 0.11)		Not allowed
			ω_0_ = 0.06 (ω_1_ = 1)	0	
M2a (selection)	4	−6407.5	*p*_0_ = 0.89		None
			*p*_1_ = 0.06 (*p*_2_ = 0.04)		
			ω_0_ = = 0.06 (ω_1_ = 1)		
			ω_2_ = 1		
M7 (beta)	2	−6349.7	*p* = 0.38, *q* = 2.68	11.6	Not allowed
M8 (beta & ω)	4	−6343.9	*p*_0_ = 0.97 (*p*_1_ = 0.03)		None
			*p* = 0.48, *q* = 4.83		
			ω_s_ = 1		

Note: Meanings for *p*, *l* and 2Δ*l* are the same with those in Table 1 and the same below in Tables 3, 4, 5.

**Table 3:** Parameter estimates and likelihood ratio statistics (2Δ*l*) for lizard sodium channel genes.

Models	*p*	*l*	Parameter estimates	2Δ*l*	PSSs
M0 (one-ratio)	1	−3720.1	ω = 0.07		None
M1a (neutral)	2	−3649.3	*p*_0_ = 0.89 (*p*_1_ = 0.11)		Not allowed
			ω_0_ = 0.05 (ω_1_ = 1)	0	
M2a (selection)	4	−3649.3	*p*_0_ = 0.89		None
			*p*_1_ = 0.06 (*p*_2_ = 0.05)		
			ω_0_ = 0.05 (ω_1_ = 1)		
			ω_2_ = 1		
M7 (beta)	2	−3591.3	*p* = 0.27, *q* = 2.43	4	Not allowed
M8 (beta & ω)	4	−3589.3	*p*_0_ = 0.96 (*p*_1_ = 0.04)		None
			*p* = 0.35, *q* = 5.04		
			ω_s_ = 1		

**Table 4:** Parameter estimates and likelihood ratio statistics (2Δ*l*) for mammalian sodium channel genes.

Models	*p*	*l*	Parameter estimates	2Δ*l*	PSSs
M0 (one-ratio)	1	−3743.6	ω = 0.07		None
M1a (neutral)	2	−3695.9	*p*_0_ = 0.93 (*p*_1_ = 0.07)		Not allowed
			ω_0_ = 0.06 (ω_1_ = 1	0	
M2a (selection)	4	−3695.9	*p*_0_ = 0.93		None
			*p*_1_ = 0.04 (*p*_2_ = 0.03)		
			ω_0_ = 0.06 (ω_1_ = 1)		
			ω_2_ = 1		
M7 (beta)	2	−3637.4	*p* = 0.36, *q* = 3.36	0	Not allowed
M8 (beta & ω)	4	−3637.4	*p*_0_ = 1 (*p*_1_ = 0)		None
			*p* = 0.36, *q* = 3.36		
			ω_s_ = 2.10		

**Table 5:** Parameter estimates and likelihood ratio statistics (2Δ*l*) for insect sodium channel genes.

Models	*p*	*l*	Parameter estimates	2Δ*l*	PSSs
M0 (one-ratio)	1	−4777.5	ω = 0.02		None
M1a (neutral)	2	−4748.3	*p*_0_ = 0.98 (*p*_1_ = 0.02)		Not allowed
			ω_0_ = 0.01 (ω_1_ = 1)	0	
M2a (selection)	4	−4748.3	*p*_0_ = 0.98		None
			*p*_1_ = 0.01 (*p*_2_ = 0.01)		
			ω_0_ = 0.01 (ω_1_ = 1)		
			ω_2_ = 1		
M7 (beta)	2	−4655.9	*p* = 0.13, *q* = 4.67	0	Not allowed
M8 (beta & ω)	4	−4655.9	*p*_0_ = 1 (*p*_1_ = 0)		None
			*p* = 0.13, *q* = 4.67		
			ω_s_ = 1.24		

## References

[b1] Catterall W. A. From ionic currents to molecular mechanisms: the structure and function of voltage-gated sodium channels. Neuron 26, 13–25 (2000). 10798388 10.1016/s0896-6273(00)81133-2

[b2] Payandeh J., Gamal El-Din. T. M., Scheuer T. Zheng N. & Catterall W. A. Crystal structure of a voltage-gated sodium channel in two potentially inactivated states. Nature 486, 135–139 (2012). 22678296 10.1038/nature11077 PMC3552482

[b3] Payandeh J., Scheuer T., Zheng N. & Catterall W. A. The crystal structure of a voltage-gated sodium channel. Nature 475, 353–358 (2011). 21743477 10.1038/nature10238 PMC3266868

[b4] Catterall W. A. Structure and function of voltaged -gated sodium channels at atomic resolution. Exp Physiol 99, 35–51 (2014). 24097157 10.1113/expphysiol.2013.071969 PMC3885250

[b5] Clare J. J., Tate S. N., Nobbs M. & Romanos M. A. Voltage-gated sodium channels as therapeutic targets. Drug Discov Today 5, 506–520 (2000). 11084387 10.1016/s1359-6446(00)01570-1

[b6] George A. L. Jr. Inherited disorders of voltage-gated sodium channels. J Clin Invest 115, 1990–1999 (2005). 16075039 10.1172/JCI25505 PMC1180550

[b7] Kass R. S. The channelopathies: novel insights into molecular and genetic mechanisms of human disease. J Clin Invest 115, 1986–1989 (2005). 16075038 10.1172/JCI26011 PMC1180558

[b8] Blumenthal K. M. & Seibert A. L. Voltage-gated sodium channel toxins: poisons, probes, and future promise. Cell Biochem Biophys 38, 215–238 (2003). 12777715 10.1385/CBB:38:2:215

[b9] Bosmans F. & Tytgat J. Voltage-gated sodium channel modulation by scorpion α-toxins. Toxicon 49, 142–158 (2007). 17087986 10.1016/j.toxicon.2006.09.023 PMC1808227

[b10] Possani L. D., Becerril B., Delepierre M. & Tytgat J. Scorpion toxins specific for Na^+^-channels. Eur J Biochem 264, 287–300 (1999). 10491073 10.1046/j.1432-1327.1999.00625.x

[b11] Gordon D. *et al.* The differential preference of scorpion α-toxins for insect or mammalian sodium channels: implications for improved insect control. Toxicon 49, 452–472 (2007). 17215013 10.1016/j.toxicon.2006.11.016

[b12] Gordon D. & Gurevitz M. The selectivity of scorpion a-toxins for sodium channel subtypes is determined by subtle variations at the interaction surface. Toxicon 41, 125–128 (2003). 12565730 10.1016/s0041-0101(02)00294-5

[b13] Zhu S., Bosmans F. & Tytgat J. Adaptive evolution of scorpion sodium channel toxins. J Mol Evol 58, 145–153 (2004). 15042334 10.1007/s00239-003-2534-2

[b14] Zhu S. *et al.* Evolutionary diversification of *Mesobuthus* α-scorpion toxins affecting sodium channels. Mol Cell Proteomics 11, M111.012054 (2012). 10.1074/mcp.M111.012054 PMC3270107 21969612

[b15] Weinberger H. *et al.* Positions under positive selection–key for selectivity and potency of scorpion α-toxins. Mol Biol Evol 27, 1025–1034 (2010). 20018978 10.1093/molbev/msp310

[b16] Sunagar K. *et al.* Evolution stings: The origin and diversification of scorpion toxin peptide scaffolds. Toxins 5, 2456–2487 (2013). 24351712 10.3390/toxins5122456 PMC3873696

[b17] Cao Z. *et al.* The genome of *Mesobuthus martensii* reveals a unique adaptation model of arthropods. Nat Commun 4, 2602 (2013). 24129506 10.1038/ncomms3602 PMC3826648

[b18] Yang Z. PAML 4: phylogenetic analysis by maximum likelihood. Mol Biol Evol 24, 1586–1591 (2007). 17483113 10.1093/molbev/msm088

[b19] Wang C. *et al.* Exploration of the functional site of a scorpion α-like toxin by site-directed mutagenesis. Biochemistry 42, 4699–4708 (2003). 12705833 10.1021/bi0270438

[b20] Karbat I. *et al.* Molecular basis of the high insecticidal potency of scorpion α-toxins. J Biol Chem 279, 31679–31686 (2004). 15133045 10.1074/jbc.M402048200

[b21] Karbat I. *et al.* The unique pharmacology of the scorpion α-like toxin Lqh3 is associated with its flexible C-tail. FEBS J 274, 1918–1931 (2007). 17355257 10.1111/j.1742-4658.2007.05737.x

[b22] Kahn R. *et al.* Molecular requirements for recognition of brain voltage-gated sodium channels by scorpion α-toxins. J Biol Chem 284, 20684–20691 (2009). 19509294 10.1074/jbc.M109.021303 PMC2742833

[b23] Rogers J. C., Qu Y., Tanada T. N., Scheuer T. & Catterall W. A. Molecular determinants of high affinity binding of α-scorpion toxin and sea anemone toxin in the S3-S4 extracellular loop in domain IV of the Na^+^ channel alpha subunit. J Biol Chem 271, 15950–15962 (1996). 8663157 10.1074/jbc.271.27.15950

[b24] Gur M. *et al.* Elucidation of the molecular basis of selective recognition uncovers the interaction site for the core domain of scorpion α-toxins on sodium channels. J Biol Chem 286, 35209–35217 (2011) 21832067 10.1074/jbc.M111.259507 PMC3186375

[b25] Wang J. *et al.* Mapping the receptor site for alpha-scorpion toxins on a Na^+^ channel voltage sensor. Proc Natl Acad Sci USA 108, 15426–15431 (2011). 21876146 10.1073/pnas.1112320108 PMC3174582

[b26] Gurevitz M. Mapping of scorpion toxin receptor sites at voltage-gated sodium channels. Toxicon 60, 502–511 (2012). 22694883 10.1016/j.toxicon.2012.03.022

[b27] Chugunov A. O. *et al.* Modular organization of α-toxins from scorpion venom mirrors domain structure of their targets, sodium channels. J Biol Chem 288, 19014–19022 (2013). 23637230 10.1074/jbc.M112.431650 PMC3696675

[b28] Pierce B. G. *et al.* ZDOCK Server: interactive docking prediction of protein-protein complexes and symmetric multimers. Bioinformatics 30, 1771–1773 (2014). 24532726 10.1093/bioinformatics/btu097 PMC4058926

[b29] Chen R. & Chung S. H. Binding modes and functional surface of anti-mammalian scorpion alpha-toxins to sodium channels. Biochemistry 51, 7775–7782 (2012). 22971116 10.1021/bi300776g

[b30] Liu L. H. *et al.* Molecular basis of the mammalian potency of the scorpion alpha-like toxin, BmKM1. FASEB J 19, 594–596 (2005). 15677695 10.1096/fj.04-2485fje

[b31] Leipold E., Lu S., Gordon D., Hansel A. & Heinemann S. H. Combinatorial interaction of scorpion toxins Lqh-2, Lqh-3, and LqhαIT with sodium channel receptor sites-3. Mol Pharmacol 65, 685–691 (2004). 14978247 10.1124/mol.65.3.685

[b32] Polis G. A. The biology of scorpions. Stanford, CA: Stanford University Press (1990).

[b33] Doron-Faigenboim A., Stern A., Mayrose I., Bacharach E. & Pupko T. Selecton: a server for detecting evolutionary forces at a single amino-acid site. Bioinformatics 21, 2101–2103 (2005). 15647294 10.1093/bioinformatics/bti259

[b34] Swanson W. J., Nielsen R. & Yang Q. Pervasive adaptive evolution in mammalian fertilization proteins. Mol Biol Evol 20, 18–20 (2003). 12519901 10.1093/oxfordjournals.molbev.a004233

[b35] Martin-Eauclaire M. F., Ferracci G., Bosmans F. & Bougis P. E. A surface plasmon resonance approach to monitor toxin interactions with an isolated voltage-gated sodium channel paddle motif. J Gen Physiol 145, 155–162 (2015). 25624450 10.1085/jgp.201411268 PMC4306711

[b36] Yang Z. & Swanson W. J. Codon-substitution models to detect adaptive evolution that account for heterogeneous selective pressures among site classes. Mol Biol Evol 19, 49–57 (2002). 11752189 10.1093/oxfordjournals.molbev.a003981

[b37] Moyle W. R. *et al.* Co-evolution of ligand-receptor pairs. Nature 368, 251–255 (1994). 8145825 10.1038/368251a0

[b38] Goh C. S., Bogan A. A., Joachimiak M., Walther D. & Cohen F. E. Co-evolution of proteins with their interaction partners. J Mol Biol 299, 283–293 (2000) 10860738 10.1006/jmbi.2000.3732

[b39] Crooks G. E., Hon G., Chandonia J. M. & Brenner S. E. WebLogo: A Sequence Logo Generator. Genome Res 14, 1188–1190 (2004). 15173120 10.1101/gr.849004 PMC419797

[b40] Armon A., Graur D. & Ben-Tal N. ConSurf: an algorithmic tool for the identification of functional regions in proteins by surface mapping of phylogenetic information. J Mol Biol 307, 447–463 (2001). 11243830 10.1006/jmbi.2000.4474

[b41] Kordis D. & Gubensek F. Adaptive evolution of animal toxin multigene families. Gene 261, 43–52 (2000). 11164036 10.1016/s0378-1119(00)00490-x

[b42] Goldin A. L. Evolution of voltage-gated Na^+^ channels. J Exp Biol 205, 575–584 (2002). 11907047 10.1242/jeb.205.5.575

[b43] Novak A. E. *et al.* Gene duplications and evolution of vertebrate voltage-gated sodium channels. J Mol Evol 63, 208–221 (2006). 16830092 10.1007/s00239-005-0287-9

[b44] Aledo J. C., Valverde H., Ruiz-Camacho M., Morilla I. & Lopez F. D. Protein-protein interfaces from cytochrome c oxidase I evolve faster than nonbinding surfaces, yet negative selection is the driving force. Genome Biol Evol 6, 3064–3076 (2014). 25359921 10.1093/gbe/evu240 PMC4255772

[b45] Nakashima K. *et al.* Accelerated evolution of *Trimeresurus flavoviridis* venom gland phospholipase A2 isozymes. Proc Natl Acad Sci USA 90, 5964–5968 (1993). 8327468 10.1073/pnas.90.13.5964 PMC46847

[b46] Duda T. F. Jr. & Palumbi S. R. Molecular genetics of ecological diversification: duplication and rapid evolution of toxin genes of the venomous gastropod *Conus*. Proc Natl Acad Sci USA 96, 6820–6823 (1999). 10359796 10.1073/pnas.96.12.6820 PMC21999

[b47] Starrett J. & Waters E. R. Positive natural selection has driven the evolution of the Hsp70s in Diguetia spiders. Biol Lett 3, 439–444 (2007). 17504732 10.1098/rsbl.2007.0159 PMC2390669

[b48] Zhu S. Positive selection targeting the cathelin-like domain of the antimicrobial cathelicidin family. Cell Mol Life Sci 65, 1285–1294 (2008). 18322645 10.1007/s00018-008-8070-x PMC11131857

[b49] Kozminsky-Atias A. & Zilberberg N. Molding the business end of neurotoxins by diversifying evolution. FASEB J 26, 576–586 (2012). 22009937 10.1096/fj.11-187179

[b50] Wigger E., Kuhn-Nentwig L. & Nentwig W. The venom optimization hypothesis: a spider injects large venom quantities only into difficult prey types. Toxicon 40, 749–752 (2002). 12175611 10.1016/s0041-0101(01)00277-x

[b51] Morgenstern D. & King G. F. The venom optimization hypothesis revisited. Toxicon 63, 120–128 (2013). 23266311 10.1016/j.toxicon.2012.11.022

[b52] Hanck D. A. & Sheets M. F. Site-3 toxins and cardiac sodium channels. Toxicon 49, 181–193 (2007). 17092528 10.1016/j.toxicon.2006.09.017 PMC1852437

[b53] Landau M. *et al.* ConSurf 2005: the projection of evolutionary conservation scores of residues on protein structures. Nucleic Acids Res 33, W299–W302 (2005). 15980475 10.1093/nar/gki370 PMC1160131

[b54] Yang Z. Likelihood ratio tests for detecting positive selection and application to primate lysozyme evolution. Mol Biol Evol 15, 568–573 (1998). 9580986 10.1093/oxfordjournals.molbev.a025957

[b55] Mintseris J. *et al.* Integrating Statistical Pair Potentials into Protein Complex Prediction. Proteins 69, 511–520 (2007). 17623839 10.1002/prot.21502

[b56] Leipold E., Hansel A., Olivera B. M., Terlau H. & Heinemann S. H. Molecular interaction of δ-conotoxins with voltage-gated sodium channels. FEBS Lett 579, 3881–3884 (2005). 15990094 10.1016/j.febslet.2005.05.077

