# Supplementary material for: Target-Driven Evolution of Scorpion Toxins
Source: Sci Rep. 2015 Oct 7;5:14973. doi: 10.1038/srep14973 (PMC4595728; doi:10.1038/srep14973)
Supplement: Supplementary Information [file srep14973-s1.pdf]

Supplementary Information

Target-Driven Evolution of Scorpion Toxins

Shangfei Zhang, Bin Gao & Shunyi Zhu

```
gi|119223235     VRDAYIAKPENCVYHCATNEGCNKLCTDNGAESGYCQWGGRYGNACWCIKLPDSVPIRVPGKCHR-
gi|20140243      VRDGYIADDKNCAYFCGRNAYCDDECKKNGAESGYCQWAGVYGNACWCYKLPDKVPIRVPGKCNGG
gi|34925420      VRDGYIADDKNCAYFCGRNAYCDDECKKKGAESGYCQWAGVYGNACWCYKLPDKVPIRVPGKCNGG
gi|270346278     VKDGYIADDRNCPYFCGRNAYCDGECKKNRAESGYCQWASKYGNACWCYKLPDDARIMKPGRCNGG
BmKM1            VRDAYIAKPHNCVYECARNEYCNDLCTKNGAKSGYCQWVGKYGNGCWCIELPDNVPIRVPGKCHR-
gi|20140244      VRDAYIAKPHNCVYECARNEYCNDLCTKNGAKSGYCQWVGKYGNGCWCKELPDNVPIRVPGKCHR-
gi|20143887      VRDAYIAKPENCVYHCATNEGCNKLCTDNGAESGYCQWGGKYGNACWCIKLPDDVPIRVPGKCHR-
BmKM4            VRDAYIAKPENCVYHCAGNEGCNKLCTDNGAESGYCQWGGRYGNACWCIKLPDDVPIRVPGKCHR-
gi|20140303      VRDGYIALPHNCAYGCLLNEFCNDLCTKNGAKIGYCNIQGKYGNACWCIELPDNVPIRVPGRCHPS
gi|3914965       VRDAYIAKPENCVYECGITQDCNKLCTENGAESGYCQWGGKYGNACWCIKLPDSVPIRVPGKCQR-
gi|1173401       VRDAYIAKPENCVYHCATNEGCNKLCTDNGAESGYCQWGGRYGNACWCIKLPDRVPIRVPGKCHR-
gi|34922454      GRDAYIAKKENCTYFCALNPYCNDLCTKNGAKSGYCQWAGRYGNACWCIDLPDKVPIRIPGPCIGR*
gi|34582353      GRDAYIADSENCTYFCGSNPYCNDLCTENGAKSGYCQWAGRYGNACWCIDLPDKVPIRIPGPCRGR*
gi|20140245      VRDAYIAQNYNCVYHCARDAYCNELCTKNGAKSGSCPYLGEHKFACYCKDLPDNVPIRVPGKCHRR
gi|47679612      VSDAYIAKPHNCVYECAKNEYCNDLCTKNGAKSGYCQWLGKYGNGCWCIELPDNVPIRVPGKCHRK
gi|56411752      VRDGYIADDKNCAYFCGRNAYCDDECKKNGAESGYCQWAGVYGNACWCYKLPDNVPIRVPGKCNGG
gi|56411748      VRDAYIAQNYNCVYHCARDAYCNELCTKNGAKSGSCPYLGEHKFACYCKDLPDNVPIRVPGKCNGG
gi|56411750      ARDAYIAQNYNCVYHCARDAYCNELCTKNGAKSGSCPYLGEHKFACYCKDLPDNVPIRVPGKCNGG
gi|75037853      VRDGYIADDKNCAYFCGRNAYCDDECKKKGAESGYCQWAGVYGNACWCYKLPDKVPIRVPGRCNGG
gi|20140246      VRDGYIADDKNCPYFCGRNAYCDDECKKNGAESGYCQWAGVYGNACWCYKLPDKVPIRVPGKCNGG
gi|20140302      GRDAYIAKNYNCVYHCFRDDYCNGLCTENGADSGYCYLAGKYGNACWCINLPDDKPIRIPGKCHRR
gi|115543218     VKDGYIADDRNCPYFCGRNAYCDEECKKNRAESGYCQWAGKYGNACWCYKLPDDARTMKPGRCNGG
gi|47679610      VSDAYIARPENCVYHCAKNEGCNKLCTDNGAESGYCQWGGRYGNACWCIKLPDDVPIRVPGKCHRK
gi|353678083     GRDAYIAQNYNCVYHCFRDDYCNGLCTENGADSGYCYLAGKYGHACWCINLPDDKPIRIPGKCHRR
gi|313930110     VKDGYIVDDKNCAYFCGRNAYCDDECEKNGAESGYCQWAGVYGNACWCYKLPDKVPIRVPGRCNG-
gi|553809561     VRDAYIAKPHNCVYECFREANCKDICTKNGAKSGYCYLFSKYGAACWCIDLPDNVPINVPGKCHPR
gi|553835062     GRDAYIAQNYNCVYHCFRDDYCNGLCTENGADSGYCYLAGKYGNACWCINLPDDKPIRIPGKCHRR
gi|553847915     VRDAYIAQNYNCLYHCARDAYCNELCTKNGAKSGSCPYLGEHKFACYCKDLPDNVPIRVPGKCHRR
Clone BmL398     VRDGYIALPHNCAYGCLLNEFCNDLCTKNGAKIGYCNIQGKYGNACWCIELPDNVPIRVPGKCHR-
```

Figure S1. Multiple sequence alignment (MSA) of the *Mesobuthus martensii* α-toxins. Apart from BmKM1 and BmKM4, two extensively studied toxins (1,2), all the other sequences are provided in their GenBank ID numbers (http://www.ncbi.nlm.nih.gov/) for avoiding confusion of their names. Clone BmL398 was isolated from a venom gland cDNA library of this scorpion species by our lab. Cysteines are shadowed in yellow and four disulfide bridges indicated by lines. Extra amino acids that will be removed after post-translational processing are underlined once and C-terminal amidation is indicated by an asterisk.

gi|699624821|*P.pubescens*	KQAFDISIMILICLNMVTMMVETDDQSEDMENILYWINLVFIVLFTGECVLKLISLRHYYFTIGWNIFDFVVVILSIVGMFLA--EMIEKYFVSPTLFRVIRLARIGRILRLIKGAKG
gi|699624906|*P.pubescens*	KQAFDIVIMVLICLNMVTMMIETDDQSKLMQDILYWINLVFVVLFTGECVFKLFSLRYYYFTIGWNIFDFVVVILSIIGMFLA--EMIEKYFVSPTLFRVIRLARIGRILRLVKGAKG
gi|699624877|*P.pubescens*	QQVFDISIMILICLNMVTMMVETDDQSEEMENILYWINLVFIVLFTGECVLKLISLRHYYFTIGWNIFDFVVVILSIVGMFLA--EMIEKYFVSPTLFRVIRLARIGRILRLIKGAKG
gi|699689265|*P.pubescens*	QQAFDIVIMMLICLNMVTMMVETDTQSKQMEDILYWINLVFVIFFTCECVLKMFALRHYYFTIGWNIFDFVVVILSIVGMFLA--EIIEKYFVSPTLFRVIRLARIGRILRLIKGAKG
gi|699649878 *P.pubescens*	KQAFDVSIMILICLNMITMMVETDDQSQEKVNILHKINMLFVAIFTGECIFKMLALRHYYFTNGWNIFDFVVVILSIVGTVLS--DIIQKYFFSPTLFRVIRLARIGRILRLIRGAKG
gi|823463918|*T.guttata*	KQAFDISIMILICLNMVTMMVETDDQSEDMENILYWINLVFIVLFTGEFVLKLISLRHYYFTIGWNIFDFVVVILSIVGMFLA--EMIEKYFVSPTLFRVIRLARIGRILRLIKGAKG
gi|823463780|*T.guttata*	QQLFDITVMVLICLNMVTMMIETDDQTELKQNILYWINFVFVVLFTGECVFKIFSLRYYYFTIGWNIFDFVVVILSIIGMFLA--EVIEKYFVSPTLFRVVRLARIGRILRLIKGAKG
gi|823473529|*T.guttata*	QQAFDIVIMMLICLNMVTMMVETDTQSKQMEDILYWINFVFVIFFTCECVLKMFALRHYYFTIGWNIFDFVVVILSIVGMFLA--EIIEKYFVSPTLFRVIRLARIGRILRLIKGAKG
gi|449491396|*T.guttata*	KQVFDITIMILICLNMVTMMVETDDQSELKTSVLYKINLVFIVIFTGECVLKMFALRHYFFTVGWNIFDFVVVILSILGIVLS--DIIEKYFVSPTLFRVIRLARIGRVLRLIRGAKG
gi|449492116|*T.guttata*	KQAFDVSIMILICLNMVTMMVETDDQSQEKVNILHKINMLFVAIFTGECIFKMLALRHYYFTNGWNIFDFVVVILSIVGTVLS--DIIQKYFFSPTLFRVIRLARIGRILRLIRGAKG
gi|699632948|*C.vociferus*	KQAFDITIMVLICLNMVTMMIETDDQSELMQNILYWINLVFVVLFTGECVFKMFSLRYYYFTIGWNIFDFVVVILSIVGMFLA--KVIEKYFVSPTLFRVVRLARIGRILRLIKGAKG
gi|699632975|*C.vociferus*	QQVFDISIMILICLNMVTMMVETDDQSKEMENILYWINLVFIVLFTGECVLKLISLRHYYFTIGWNIFDFVVVILSIVGMFLA--EMIEKYFVSPTLFRVIRLARIGRILRLIKGAKG
gi|699677371|*C.vociferus*	KQAFDVSIMILICLNMVTMMVETDDQSQEKVNILHKINMLFVAIFTGECIIKMLALRHYYFTNGWNIFDFVVVILSIVGTVLS--DIIQKYFFSPTLFRVIRLARIGRILRLIRGAKG
gi|699659219|C.vociferus	KQVFDITIMILICLNMVTMMVETDDQSELKTSVLYKINLVFIVIFTGECVLKMFALRYYYFTIGWNIFDFVVVILSILGIVLS--DIIEKYFVSPTLFRVIRLARIGRVLRLIRGAKG
gi|543245193|*G.fortis*	KQAFDISIMILICLNMVTMMVETDDQSEEMENILYWINLVFIVLFTGEFVLKLISLRHYYFTIGWNIFDFVVVILSIVGMFLA--EMIEKYFVSPTLFRVIRLARIGRILRLIKGAKG
gi|543298808|*G.fortis*	KQVFDITIMILICLNMVTMMVETDDQSELKTSVLYKINLVFIVIFTGECVLKMFALRHYFFTVGWNIFDFVVVILSILGIVLS--DIIEKYFVSPTLFRVIRLARIRRLRRLGRGACT
gi|647570007|*G.gallus*	KQAFDIGIMVLICLNMVTMMIETDDQSELMQNILYWINFVFVVLFTGECVLKLFSLRYYYFTVGWNIFDFVVVILSIVGMFLA--KVIEKYFVSPTLFRVIRLARIGRILRLIKGAKG
gi|342837673|*G.gallus*	KQVFDITIMILICLNMVTMMVETDDQSETKTDILYKINLIFIVIFTGECVLKMFALRYYYFTIGWNIFDFVVVILSIAGIVLS--DIIEKYFVSPTLFRVIRLARIGRVLRLIRGAKG
gi|543348889|*P.humilis*	KQVFDISIMILICLNMVTMMVETDDQSKEMETILSRINLVFIVLFTGEFVLKLISLRHYYFTVGWNIFDFVVVILSIVGMFLA--EMIEKYFVSPTLFRVIRLARIGRILRLIKGAKG
gi|541981351|*F.cherrug*	KQAFDIAIMVLICLNMVTMMIETDDQSDLMQNILYWINLVFVVLFTGECVFKMFSLRYYYFTIGWNIFDFVVVILSIVGMFLA--KVIEKYFVSPTLFRVVRLARIGRILRLIKGAKG
gi|541971530|*F.cherrug*	KQVFDITIMILICLNMVTMMVETDDQSELKTSVLYMINLVFIVIFTGECVLKMLALRYYYFTIGWNIFDFVVVILSILGIVLS--DIIEKYFVSPTLFRVIRLARIGRVLRLIRGAKG
gi|696965978|*C.canorus*	KQAFDITIMVLICLNMVTMMIETDDQGQLMQDILYWINLVFVVLFTGECILKMFSLRYYYFTIGWNIFDFVVVILSIIGMFLA--DVIEKYFVSPTLFRVVRLARIGRILRLIKGAKG
gi|696965919|*C.canorus*	KQVFDISIMILICLNMVTMMVETDDQSKEMEAILSWINLVFIILFTGEFVLKLISLRHYYFTIGWNIFDFVVVILSIVGMFLA--EMIEKYFVSPTLFRVIRLARIGRILRLIKGAKG
gi|696965965|*C.canorus*	QQVFDISIMILICLNMVTMMVETDDQSKEMENILSWINLVFIVLFTGECVLKLISLRHYYFTIGWNIFDFVVVILSIVGMFLA--EMIEKYFVSPTLFRVIRLARIGRILRLIKGAKG
gi|696998543|*C.canorus*	KQVFDITIMILICLNMVTMMVETDDQSELKTSILYKINLVFIVIFTGECVLKMFALRYYYFTIGWNIFDFVVVILSILGIVLS--DIIEKYFVSPTLFRVIRLARIGRVLRLIRGAKG
gi|683906241|*S.canaria*	KQLFDIIIMVLICLNMVTMMIETDDQNELMQNILYWINLVFVVLFTGECVFKIFSLRYYYFTIGWNIFDFVVVILSIIGMFLA--EVIEKYFVSPTLFRVVRLARIGRILRLIKGAKG
gi|669281868|*C.brachyrhynchos*	KQVFDITIMILICLNMVTMMVETDDQSELKTSVLYKINLVFIVIFTGECVLKMFALRYYYFTVGWNIFDFVVVILSILGIVLS--DIIEKYFVSPTLFRVIRLARIGRVLRLIRGAKG
gi|669281061|*C.brachyrhynchos*	RQLFDITVMVLICLNMVTMMIETDDQTELMQNILYWINLVFVVLFTCECVFKIFSLRYYYFTIGWNIFDFVVVILSIIGMFLA--EVIEKYFVSPTLFRVVRLARIGRILRLIKGAKG
gi|726994909|*C.cornix*	KQVFDITIMILICLNMVTMMVETDDQSELKTSVLYKINLVFIVIFTGECVLKMFALRYYYFTVGWNIFDFVVVILSILGIVLS--DIIEKYFVSPTLFRVIRLARRRRGMRLIRGAKG
gi|719771801|*T.guttatus*	KQAFDISIMILICLNMVTMMVETDDQSKEMENILYWINLVFIVLFTGEFVLKLISLRHYYFTIGWNIFDFVVVILSIVGMFLA--EMIEKYFVSPTLFRVIRLARIGRILRLIKGAKG
gi|719770223|*T.guttatus*	QQVFDISIMILICLNMVTMMVETDDQSDEMENILYWINLVFIVLFTGECVLKLISLRHYYFTIGWNIFDFVVVILSIVGMFLA--EMIEKYFVSPTLFRVIRLARIGRILRLIKGAKG
gi|719776079|*T.guttatus*	QQAFDIVIMMLICLNMVTMMVETDTQSKQMENILYWINLVFVIFFTCECVLKMFALRHYYFTIGWNIFDFVVVILSIVGMFLA--EIIEKYFVSPTLFRVIRLARIGRILRLIKGAKG
gi|719730318|*T.guttatus*	KQVFDITIMILICLNMVTMMVETDDQSQLKTDVLYKVNLVFIVIFTGECVLKMFALRYYYFTIGWNIFDFVVVILSIVGIVLS--DIIEKYFVSPTLFRVIRLARIGRVLRLIRGAKG
gi|719791574|*T.guttatus*	KQAFDVSIMILICLNMVTMMVETDDQSQEKVNILHKINMLFVAIFTGECIIKMVALRHYYFTNGWNIFDFVVVILSIVGTVLS--DIIQKYFFSPTLFRVIRLARIGRILRLIRGAKG
gi|543743408|*C.livia*	KQIFDVSIMILICLNMVTMMVETDDQSQEKVNILHKINMLFVAIFTGECIIKMLALRHYYFTNGWNIFDFVVVILSIVGTVLS--DIIQKYFFSPTLFRVIRLARIGRILRLIRGAKG
gi|697486596|*S.camelus*	KQVFDISIMILICLNMVTMMVETDDQSKEMETILSRINLVFIILFTGECVLKLISLRHYYFTIGWNIFDFVVVILSIVGMFLA--EMIEKYFVSPTLFRVIRLARIGRILRLIKGAKG
gi|697486691|*S.camelus*	KQAFDISIMVLICLNMVTMMIETDDQSELMQNILYWINLIFVVLFTGECVLKLFSLRYYYFTIGWNIFDFVVVILSIVGMFLA--KVIEKYFVSPTLFRVVRLARIGRILRLIKGAKG
gi|697513240|*S.camelus*	KQVFDITIMILICLNMVTMMVETDDQSQFKTDVLYKVNLVFIVIFTGECVLKMFALRYYYFTIGWNIFDFVVVILSIVGIVLS--DIIEKYFVSPTLFRVIRLARIGRVLRLIRGAKG
gi|690446304|*P.adeliae*	KQAFDITIMVLICLNMVTMMIETDDQGELMQNILYWINLVFVVLFAGECVFKLFSLRYYYFTIGWNIFDFVVVILSVVGMFLA--KVIEKYFVSPTLFRVVRLARIGRILRLIKGAKG
gi|690439493|*P.adeliae*	KQAFDVSIMILICLNMVTMMVETDDQSQEKVNILHKINMLFVAVFTGECIIKMLALRHYYFTNGWNIFDFVVVILSIVGTVLS--DIIQKYFFSPTLFRVIRLARIGRILRLIRGAKG

gi|690428680|*P.adeliae*	KQVFDITIMILICLNMVTMMVETDDQSELKTSVLYKINLVFIVIFTGECLLKMFALRYYYFTIGWNIFDFVVVILSILGIVLS--DIIEKYFVSPTLFRVIRLARIGRVLRLIRGAKG
gi|514743636|*A.platyrhynchos*	KQAFDISIMVLICLNMVTMMIETDDQGDLMQNILYWINLVFVVLFTGECVFKLFSLRYYYFTIGWNIFDFVVVILSIVGMFLA--KVIEKYFVSPTLFRVVRLARIGRILRLIKGAKG
gi|514743648|*A.platyrhynchos*	QQAFDISIMILICLNMVTMMVETDDQSKEMENILYWINLVFIVLFTGECVLKLISLRHYYFTIGWNIFDFVVVILSIVGMFLA--EMIEKYFVSPTLFRVIRLARIGRILRLIKGAKG
gi|514755303|*A. platyrhynchos*	KQVFDITIMILICLNMVTMMVETDDQSELKTDILYKINLVFIVIFTGECVLKMFALRYYYFTIGWNIFDFVVVILSIAGIVLS--DIIEKYFVSPTLFRVIRLARIGRVLRLIRGAKG
gi|729721552|*H. leucocephalus*	KQAFDITIMVLICLNMVTMMIETDGQGELMQNILYWINLVFVVLFTGECVLKLFSLRYYYFTVGWNIFDFVVVILSIVGMFLA--KVIEKYFVSPTLFRVVRLARIGRILRLIKGAKG
gi|701390492| *C. pelagica*	KQAFDITIMVLICLNMVTMMIETDDQGELMQNILYWINLVFVVLFTGECILKLFSLRYYYFTIGWNIFDFVVVILSIVGMFLA--EVIKKYFVSPTLFRVVRLARIGRILRLIKGAKG
gi|701375204|*C. pelagica*	QQAFDIVIMMLICLNMVTMMVETDTQSKKMEDILYWINFVFVIFFTCECVLKMFALRHYYFTIGWNIFDFVVVILSIVGMFLA--EIIEKYFVSPTLFRVIRLARIGRILRLIKGAKG
gi|701406438|*C. pelagica*	KQVFDITIMILICLNMVTMMVETDDQSELKTSVLYKINLVFIVIFTGECVLKMFALRHYYFTIGWNIFDFVVVILSILGIVLS--DIIEKYFVSPTLFRVIRLARIGRVLRLIRGAKG
gi|705688749|*C. macqueenii*	KQAFDITIMVLICLNMVTMMIETDDQGELMKSILYWINLVFVVLFTGECVLKLFSLRYYYFTIGWNIFDFVVVILSIVGMFLA--KVIEKYFVSPTLFRVVRLARIGRILRRIQGAKV
gi|705668534|*C. macqueenii*	QQVFDISIMILICLNMVTMMVETDDQSKEMETILYWINLVFIVLFTGECVLKLISLRHYYFTIGWNIFDFVVVILSIVGMFLA--EMIEKYFVSPTLFRVIRLDRIGRILRLIKGAKG
gi|705671331|*C. macqueenii*	KQVFDITIMILICLNMVTMMVETDDQSDLKTSVLYKINLVFIVIFTGECVLKMFALRYYYFTIGWNIFDFVVVILSILGIVLS--DIIEKYFVSPTLFRVIRLARIGRVLRLIRGAKG
gi|705695710|*C. macqueenii*	KQAFDVSIMILICLNMVTMMVETDDQSQEKVNILHKINMLFVAIFTGECIIKMLALRHYYFTNGWNIFDFVVVILSIVGTVLS--DIIQKYFFSPTLFRVIRLARIGRVLRLIRGAKG
gi|527269695|*M. undulatus*	KQAFDISIMILICLNMVTMMVETDDQSEDMENILYWINLVFIVLFTGEFVLKLISLRHYYFTVGWNIFDFVVVILSIVGMFLA--EMIEKYFVSPTLFRVIRLARIGRILRLIKGAKG
gi|527269392|*M. undulatus*	KQVFDISIMILICLNMVTMMVETDDQSKEMETILSRINLVFIILFTGECVLKLISLRHYYFTLGWNIFDFVVVILSIVGMFLA--EMIEKYFVSPTLFRVIRLARIGRILRLIKGAKG
gi|527269353|*M. undulatus*	KQEFDITIMVLICINMITMMIETDDQAELMQNILYWINLAFVVLFTGECVLKLFSLRYYYFTIGWNIFDFVVVILSIVGMFLA--KVIEKYFVSPTLFRVIRLARIGRILRLIRGAKG
gi|700400984|*O. hoazin*	KQAFDVSIMILICLNMITMMVETDDQSQEKVNILHKINMLFVAIFTGECIIKMLALRHYYFTNGWNIFDFVVVILSIVGTVLS--DIIQKYFFSPTLFRVIRLARIGRVLRLIRGAKG
gi|700375357|*O. hoazin*	KQVFDITIMILICLNMITMMVETDDQSELKTSVLYKINLVFIVIFTGECVLKMFALRYYYFTIGWNIFDFVVVILSILGIVLS--DIIEKYFVSPTLFRVIRLARIGRVLRLIRGAKG
gi|663239565|*C. anna*	KQVFDISIMILICLNMVTMMVETDDQSKEMETILSRINLVFIILFTGEFVLKLISLRHYYFTVGWNIFDFVVVILSIVGMFLA--EMIEKYFVSPTLFRVIRLARIGRILRLIKGAKG
gi|663288339|*C. anna*	KQVFDITIMILICLNMVTMMVETDDQSELKTAVLYKINLVFIVIFTGECVLKMFALRHYFFTIGWNIFDFVVVILSILGIVLS--DIIEKYFVSPTLFRVIRLARIGRVLRLIRGAKG
gi|675430434|*M. vitellinus*	KQVFDISIMILICLNMVTMMVETDDQSKEMETILSRINLVFIILFTGEFVLKLISLRHYYFTIGWNIFDFVVVILSIVGMFLA--EMIEKYFVSPTLFRVIRLARIGRILRLIKGAKG
gi|675407061|*M. vitellinus*	KQVFDITIMILICLNMVTMMVETDDQSELKTSVLYKINLVFIVIFTGECVLKMFALHYHFFTVGWNIFDFVVVILSILGIVLS--DIIEKYFVSPTLFRVIRLARIGRVLRLIRGAKG
gi|704177515|*B. rhinoceros*	KQVFDISIMILICLNMVTMMVETDDQSKEMEIILSRINLVFIILFTGEFVLKLISLRHYYFTIGWNIFDFVVVILSIVGMFLA--EMIEKYFVSPTLFRVIRLARIGRILRLIKGAKG
gi|704199054|*B. rhinoceros*	KQAFDITIMVLICLNMVTMMIETDDQGERMQDILYWINLVFVVLFTGECVFKMFSLRCYYFTVGWNIFDFVVVILSIIGTCLD--EMIQKYFVSPTLFRVIRLARIGRILRLIKGAKG
gi|704169897|*B. rhinoceros*	KQVFDITIMILICLNMVTMMVETDDQSEFKTSILNKINLVFIVIFTGECVLKMFALRYYYFTIGWNIFDFVVVIFSILGIVLS--DIIEKYFVSPTLFRVIRLARIGRVLRLIRGAKG
gi|542150274|*Z. albicollis*	KQVFDISIMILICLNMVTMMVETDDQSKEMETILSRINLVFIVLFTGEFVLKLISLRHYYFTIGWNIFDFVVVILSIVGMFLA--EMIEKYFVSPTLFRVIRLARIGRILRLIKGAKG
gi|733922787|*M. gallopavo*	KQVFDITIMILICLNMVTMMVETDDQSETKTDILYKINLVFIVIFTGECVLKMFALRYYFFTIGWNIFDFVVVILSIAGIVLS--DIIEKYFVSPTLFRVIRLARIGRVLRLIRGAKG
gi|733891677|*M. gallopavo*	NQAFDIAIMILICLNMITMMVETYEQSDTKTNVLNKINILFVAIFTTECILKLVALRQYYFSNAWNIFDIVVVIMSIVALLLSGIGKAFEHFLPPTLFRVIRLARIGRILRLIRAAKG
gi|686594368|*A. forsteri*	KQAFDISIMILICLNMVTMMVETDDQSEDMENILYWINLVFIVLFTGECVLKLISLRHYYFTIGWNIFDFVVVIISIVGMFLA--EMIEKYFVSPTLFRVIRLARIGRILRLIKGAKG
gi|686594360|*A. forsteri*	QQVFDISIMILICLNMVTMMVETDDQSKEMENILYWINLVFIVLFTGECVLKLISLRHYYFTLGWNIFDFVVVILSIVGMFLA--EMIEKYFVSPTLFRVSSDSRIGRILRLIKGAKG
gi|704329645|*C. carolinensis*	KQAFDITIMVLICLNMVTMMIETDDQSEMMQHILYWINLVFVVLFTGECVFKLFSLRYYYFTVGWNIFDFVVVILSIVGMFLA--KVIEKYFVSPTLFRVVRLARIGRILRLIKGAKG
gi|704298804|*C. carolinensis*	KQVFDITIMILICLNMVTMMVETDDQSEAKTSVLYKINLVFIVIFTGECVLKMFALRYYYFTIGWNIFDFVVVILSILGIVLS--DIIEKYFVSPTLFRVIRLARIGRVLRLIRGAKG
gi|683461587|*C. carolinensis*	RQAFDVVIMILICLNMISMMVETYEQSETKSNILNKINMLFVALFTTECVLKLVALRQYYFSNAWNIFDLVVVIMSLVALLLSGIGKAFEHFLPPTLFRVIRLARIGRILRLIRAARG
gi|699582281|*A. vittatum*	QQAFDIVIMMLICLNMVTMMVETDTQSEQMEDILYWINLVFVIFFTCECVLKMFALRHYYFTIGWNIFDFVVVILSIVGMFLA--EIIEKYFVSPTLFRVIRLARIGRILRLIKGAKG
gi|699600245|*A. vittatum*	KQVFDITIMILICLNMVTMMVETDDQSELKTSVLYKINLVFIVIFTGECVLKMFALRYYFFTIGWNIFDFVVVILSILGIVLS--DIIEKYFVSPTLFRVIRLARIGRVLRLIRGAKG
gi|723125282|*P. lepturus*	KQAFDITIMVLICLNMVTMMIETDDQGELMQNILYWINLVFVVLFTGECVLKLFSLRYYYFTIGWNIFDFVVVILSIVGMFLA--EVIEKYFVSPTLFRVVRLARIGRILRLIKGAKG
gi|678145895|*T. erythrolophus*	QQVFDISIMILICLNMVTMMVETDDQSKEMETILYWINLVFIVLFTGECVLKLISLRHYYFTIGWNIFDFVVVILSIVGMFLA--EMIEKYFVSPTLFRVIRLARIGRILRLIKGAKG
gi|698441979|*G. stellata*	KQAFDITIMVLICLNMVTMMIETDDQGELMQNILYWINLVFVVLFTGECVFKLFSLRYYYFTIGWNIFDFVVVILSIVGMFLA--KVIEKYFVSPTLFRVVRLARIGRILRLIKGAKG
gi|697017762|*F. glacialis*	KQAFDITIMVLICLNMVTMMIETDDQGELMQNILYWINLVFVVLFTGECVFKLFSLRYYYFTIGWNIFDFVVVILSIVGMFLA--EMIEKYFVSPMLFRVIRLARIGRILRLIKGAKG
gi|698407580|*C. cristata*	KQAFDITIMVLICLNMVTMMIETDDQGELMQNILYWINLVFVVLFTGECVFKLFSLRYYYFTIGWNIFDFVVVILSIIGMFLA--EVIEKYFVSPTLFRVIRLARIGRILRLIKGAKG
gi|677983296|*A. chloris*	KQVFDITIMILICLNMVTMMVETDDQSELKTSVLYQINLVFIVVFTGECVLKMFALRYYYFTVGWNIFDFVVVIFSILGIVLS--DIIEKYFVSPTLFRVIRLARIGRVLRLIRGAKG

gi|677465800|*N. notabilis*	KQVFDIVIMILICLNMVTMMVETDDQSELKTSVLYKINLVFIVIFTGECVLKMFALRHYFFTIGWNIFDFVVVILSILGIVLS--DIIEKYFVSPTLFRVIRLARIGRVLRLIRGAKG
gi|677394262|*L. discolor*	KQAFDVSIMILICLNMITMMVETDDQSQEKVNILHKINMLFVAIFTGECIIKMLALRHYYFTNGWNIFDFVVVILSIVGTVLS--DIIQKYFFSPTLFRVIRLARIGRILRLIRGAKG
gi|723551436|*B. regulorum*	KQVFDIIIMILICLNMVTMMVETDDQSELKTSVLYKINLVFIVIFTGECVLKMFALRYYYFTIGWNIFDFVVVILSILGIVLS--DIIEKYFVSPTLFRVIRLARIGRVLRLIRGAKG
gi|678995589|*E. helias*	KQAFDITVMVLICLNMVTMMIETDDQGELMERILYWINLVFVVLFTGECVFKMFSLRCYYFTIGWNVFDFVVVILSIVGMFLA--KVIEEYFVSPTLFRVVRLARIGRILRLIKGAKG

Figure S2. MSA of voltage-sensor domain (VSD) (DIV) of bird Na⁺ channels. Loops involved in interaction with α-toxins are boxed in orange where three sites responsible for binding to Lqh2, an α-toxin from *Leiurus quinquestriatus hebraeus* (3), are shadowed in yellow.

gi|343098400|*A. carolinensis*	KQAFDISIMILICLNMVTMMVETDDQTDAMETNLYRINLIFIVLFTGECVLKLISLRYYYFTIGWNIFDFVVVILSIVGMFLAEIIEKYFVSPTLFRVIRLARIGRILRLIKGAKG
gi|343098402|*A. carolinensis*	KQAFDISIMILICLNMVTMMVETDDQSKEMETILSRINLVFIILFTGECVLKLTSLRHYYFTIGWNIFDFVVVILSIVGMFLAEIIEKYFVSPTLFRVIRLARIGRILRLIKGAKG
gi|637324084|*A. carolinensi*	RQVFDISIMILICLNMITMMVETDDQSQQTEEILYRINLIFIVLFTGECVLKLISLRYYYFTIGWNIFDFVVVILSIVGMFLAEIIEKYFVSPTLFRVIRLARIGRILRLIKGAKG
gi|641451020|*A. carolinensis*	QQAFDILIMLLICLNMVTMMVETDTQSKQMEEILYWINLVFVIFFTCECVLKMFALRHYYFTIGWNIFDFVVVILSIVGMFLAEIIEKYFVSPTLFRVIRLARIGRILRLIKGAKG
gi|343098410|*A. carolinensis*	RQAFDIGIMSLICLNMVTMMVETDDEDESKKKILYWINVIFIILFTGEFLLKLIALRYYYFTIGWNIFDFVVVILSFIGMFLSELIEKYFVSPTLFRVIRLARIGRILRLIKGAKG
gi|343098406|*A. carolinensis*	NQAFDVCIMLLICLNMITMMVETDDQSPEKVNILYKINMVFVAIFTAECISKLVALRHYYFTNGWNIFDFVVVILSIVGSVLSDIIQKYFFSPTLFRVIRLARIGRILRLIRGAKG
gi|343098404|*A. carolinensis*	QQAFDIVIMILICLNMVTMMVETDDQSQTKIDILFQINLIFIVIFTTECFLKMIALRYYFFTVGWNIFDFVVVILSIAGLVLSDLIEKYFVSPTLFRVIRLARIGRVLRLIRGAKG
gi|675822220|*T. sirtalis*	KQAFDISIMILICLNMVTMMVETDDQTDAMETILYRINFIFIVLFTGECVLKLISLRYYYFTIGWNIFDFVVVILSIVGMFLAEIIEKYFVSPTLFRVIRLARIGRILRLIKGAKG
gi|675822222|*T. sirtalis*	KQAFDITIMILICLNMVTMMVETDDQSKEMEIILSRINLVFIILFTGECVLKLISLRHYYFTIGWNIFDFVVVILSIVGMFLAEIIEKYFVSPTLFRVIRLARIGRILRLIKGAKG
gi|675822218|*T. sirtalis*	QQVFDISIMILICLNMITMMVETDDQSKQTEDILYRINFIFIVLFTGECVLKLISLRYYYFTIGWNIFDFVVVILSIVGMFLAEIIEKYFVSPTLFRVIRLARIGRILRLIKGAKG
gi|675822226|*T. sirtalis*	QQAFDIVIMLLICLNMVTMMVETDTQSKQMEEILYWINFVFVIFFTCECVLKMFALRHYYFTIGWNIFDFVVVILSVVGMFLAEIIEKYFVSPTLFRVIRLARIGRILRLIKGAKG
gi|675822228|*T. sirtalis*	KQAFDIGIMILICLNMITMMVETADQDTSVEDILYWINLIFIVLFTAECLLKLIALRYYYFTIGWNIFDFVVVIFSIVGMCLSKIIEKFFVSPTLFRVVRLARIGRILRLIKGAKG
gi|60265593|*T. sirtalis*	QQAFDIIIMILICLNMVTMMVETDDQSQTKITILAQINLVFIIIFTSECFLKMIALRHYFFTNGWNIFDFVVVILSIVGLVLSDIIEKYFVSPTLFRVIRLARIGRVLRLIRGAKG
gi|60265591|*T. sirtalis*	QQAFDIIIMILICLNMVTMMVETDDQSQTKITILAQINLVFIIIFTSECLLKMIALRHYFFTNGWNIFDFVVVILSIVGLVLSDIIEKYFVSPTLFRVIRLARIGRALRLIRGAKG
gi|602644140|*P. bivittatus*	KQAFDISIMILICLNMVTMMVETDDQSKEMEIILSRINLVFIVLFTGECILKLISLRHYYFTIGWNIFDFVVVILSIVGMFLAEIIEKYFVSPTLFRVIRLARIGRILRLIKGAKG
gi|602651607|*P. bivittatus*	RQVFDISIMILICLNMITMMVETDDQSKQTEDILYRINLIFIVLFTGECVLKLISLRYYYFTIGWNIFDFVVVILSIVGMFLAEIIEKYFVSPTLFRVIRLARIGRILRLIKGAKG
gi|602629712|*P. bivittatus*	QQAFDIIIMLLICLNMVTMMVETDTQSKQMEEILYWINFVFVIFFTCECVLKMFALRHYYFTIGWNIFDFVVVILSIVGMFLAEIIEKYFVSPTLFRVIRLARIGRILRLIKGAKG
gi|602669993|*P. bivittatus*	HQLFDIAIMTLICLNMVTMMVEADDQSPEKTYVLNKINILFITLFTTECAMKLVALRHYYFINGWNIFDLVVVIMSIVGNVLSGIVVS-F-SPTLFRVIRLVRIGRILRLIRSARG
gi|602642869|*P. bivittatus*	NQAFDVCIMLLICLNMVTMMVETDDQSPEKVNILYRINMVFVAIFTAECIFKMTALRHYYFTNGWNIFDFVVVILSIVGSVLSDIIQKYFFSPTLFRVIRLARIGRILRLIRGAKG
gi|602636073|*P. bivittatus*	QQAFDIVIMILICLNMVTMMVETDDQSQTKINILAQINLIFIIIFTSECFLKMIALRYYFFTNGWNIFDFVVVILSIVGLVLADIIEKYFVSPTLFRVIRLARIGRVLRLIRGAKG
gi|602672446|*P. bivittatus*	RQAFDIGIMVLICLNMVTMMVETADQDSSVEEILYWINLIFVVIFTGECLLKLIALRYYYFTIGWNIFDFVVVIFSIVGMCLSQIIEKFFVSPTLFRVVRLARIGRVLRLIKGAKG
gi|565318632|*O. hannah*	KQAFDISIMILICLNMVTMMVETDDQSKEMEIILSRINLVFIILFTGECILKLISLRHYYFTIGWNIFDFVVVILSIVGMFLAEIIEKYFVSPTLFRVIRLARIGRILRLIKGAKG
gi|565312590|*O.* hannah	QQAFDILIMLLICLNMVTMMVETDTQSKQMEDILYWINFVFVIFFTCECVLKMFALRHYYFTIGWNIFDFVVVILSIVGMFLAEIIEKYFVSPTLFRVIRLARIGRILRLIKGAKG
gi|565307265|*O. hannah*	KQAFDIGIMILICLNMVTMMVETADQDSSVEEILYWINLFFIVIFTGECLLKLIALRYYYFTIGWNIFDFVVVIFSIVGMCLSQIIEKFFVSPTLFRVVRLARIGRVLRLIKGAKG
gi|565321537|*O. hannah*	QQVFDISIMILICLNMITMMVETDDQSKKTEDILYRINFIFIVLFTGECVLKLISLRYYYFTIGWNIFDFVVVILSIVGMFLAEIIEKYFVSPTLFRVIRLARIGRILRLIKGAKG
gi|565321905|*O. hannah*	KQAFELFIISLIFLNVVVMAVEYEGQDVAADQLLEKINCVFVALFTGECVMKLLALRLYFFKDSWNIFDLVVVILSIISLAATQLWQRLNFPPTILRVIRVIRISRLLRLIRGARG
gi|253910955|*T. couchii*	QQAFDIIIMILICLNMVTMMVETDDQSQTKINILAQINLVFIIIFTSECFLKMIALRHYFFTNGWNIFDFVVVILSIVGLVLSDIIEKYFVSPTLFRVIRLARIGRVLRLIRGAKG
gi|253910953|*T. elegans*	QQAFDIVIMILICLNMVTMMVETDDQSQTKINILAQINLVFIIIFTSECFLKMIALRHYFFTNGWNIFDFVVVILSIVGLVLSDIIEKYFVSPTLFRVIRLARIGRVLRLIRGAKG

Figure S3. MSA of VSD (DIV) of lizard Na⁺ channels. Sequence annotations are the same with those in Fig. S2 (the same below).

```
rNav1.2    KQVFDISIMILICLNMVTMMVETDDQSQEMTNILYWINLVFIVLFTGECVLKLISLRHYYFTIGWNIFDFVVVILSIVGMFLAELIEK--YFVSPTLFRVIRLARIGRILRLIKGAKG
rNav1.1    RQVFDISIMILICLNMVTMMVETDDQSDYVTSILSRINLVFIVLFTGECVLKLISLRHYYFTIGWNIFDFVVVILSIVGMFLAELIEK--YFVSPTLFRVIRLARIGRILRLIKGAKG
rNav1.3    RQVFDISIMILICLNMVTMMVETDDQSKYMTLVLSRINLVFIVLFTGEFLLKLISLRYYYFTIGWNIFDFVVVILSIVGMFLAELIEK--YFVSPTLFRVIRLARIGRILRLIKGAKG
rNav1.4    KQVFDISIMILICLNMVTMMVETDDQSQLKVDILYNINMVFIIIFTGECVLKMFALRHYYFTIGWNIFDFVVVILSIVGLALSDLIQK--YFVSPTLFRVIRLARIGRVLRLIRGAKG
rNav1.5    KQAFDVTIMFLICLNMVTMMVETDDQSPEKVNILAKINLLFVAIFTGECIVKMAALRHYYFTNSWNIFDFVVVILSIVGTVLSDIIQK--YFFSPTLFRVIRLARIGRILRLIRGAKG
rNav1.6    QQAFDIVIMMLICLNMVTMMVETDTQSKQMENILYWINLVFVIFFTCECVLKMFALRHYYFTIGWNIFDFVVVILSIVGMFLADIIEK--YFVSPTLFRVIRLARIGRILRLIKGAKG
rNav1.7    NQAFDITIMVLICLNMVTMMVEKEGQTEYMDYVLHWINMVFIILFTGECVLKLISLRHYYFTVGWNIFDFVVVILSIVGMFLAEMIEK--YFVSPTLFRVIRLARIGRILRLIKGAKG
rNav1.8    RQAFDIIIMVLICLNMITMMVETDEQGEEKTKVLGRINQFFVAVFTGECVMKMFALRQYYFTNGWNVFDFIVVILSIGSLLFSAILKSLENYFSPTLFRVIRLARIGRILRLIRAAKG
rNav1.9    SQIFDIIIISLIILNMISMMAESYNQPKAMKSILDHLNWVFVVIFTLECLIKIFALRQYYFTNGWNLFDCVVVLLSIVSTMISTLENQEHIPFPPTLFRIVRLARIGRILRLVRAARG
mNav1.2    KQVFDISIMILICLNMVTMMVETDDQSQEMTNILYWINLVFIVLFTGECVLKLISLRHYYFTIGWNIFDFVVVILSIVGMFLAELIEK--YFVSPTLFRVIRLARIGRILRLIKGAKG
mNav1.1    RQVFDISIMILICLNMVTMMVETDDQSDYVTSILSRINLVFIVLFTGECVLKLISLRHYYFTIGWNIFDFVVVILSIVGMFLAELIEK--YFVSPTLFRVIRLARIGRILRLIKGAKG
mNav1.3    RQVFDISIMILICLNMVTMMVETDDQSKYMTLVLSRINLVFIVLFTGEFLLKLISLRYYYFTIGWNIFDFVVVILSIVGMFLAELIEK--YFVSPTLFRVIRLARIGRILRLIKGAKG
mNav1.4    KQVFDISIMILICLNMVTMMVETDDQSQLKVDILYNINMVFIIVFTGECVLKMFALRHYYFTIGWNIFDFVVVILSIVGLALSDLIQK--YFVSPTLFRVIRLARIGRVLRLIRGAKG
mNav 1.5   KQAFDVTIMFLICLNMVTMMVETDDQSPEKVNILAKINLLFVAIFTGECIVKMAALRHYYFTNSWNIFDFVVVILSIVGTVLSDIIQK--YFFSPTLFRVIRLARIGRILRLIRGAKG
mNav 1.6   QQAFDIVIMMLICLNMVTMMVETDTQSKQMENILYWINLVFVIFFTCECVLKMFALRHYYFTIGWNIFDFVVVILSIVGMFLADIIEK--YFVSPTLFRVIRLARIGRILRLIKGAKG
mNav1.7    NQAFDITIMVLICLNMVTMMVEKEGQTDYMSFVLYWINVVFIILFTGECVLKLISLRHYYFTVGWNIFDFVVVILSIVGMFLAEMIEK--YFVSPTLFRVIRLARIGRILRLIKGAKG
mNav1.8    RQAFDIIIMALICLNMITMMVETDNQSEEKTKVLGRINQFFVAVFTGECVMKMFALRQYYFTNGWNVFDFIVVILSISSLLFSAILSSLESYFSPTLLRVIRLARIGRILRLIRAAKG
mNav1.9    SQVFDVIILGLIVTNMIIMMAESEGQPNEVKKIFDILNIVFVVIFTVECLIKVFALRQHYFTNGWNLFDCVVVVLSIISTLVSGLENS--NVFPPTLFRIVRLARIGRILRLVRAARG
hNav1.2    KQVFDISIMILICLNMVTMMVETDDQSQEMTNILYWINLVFIVLFTGECVLKLISLRYYYFTIGWNIFDFVVVILSIVGMFLAELIEK--YFVSPTLFRVIRLARIGRILRLIKGAKG
hNav1.1    RQVFDISIMILICLNMVTMMVETDDQSEYVTTILSRINLVFIVLFTGECVLKLISLRHYYFTIGWNIFDFVVVILSIVGMFLAELIEK--YFVSPTLFRVIRLARIGRILRLIKGAKG
hNav1.3    RQVFDISIMILICLNMVTMMVETDDQGKYMTLVLSRINLVFIVLFTGEFVLKLVSLRHYYFTIGWNIFDFVVVILSIVGMFLAEMIEK--YFVSPTLFRVIRLARIGRILRLIKGAKG
hNav1.4    KQAFDITIMILICLNMVTMMVETDDQSQLKVDILYNINMIFIIIFTGECVLKMLALRQYYFTVGWNIFDFVVVILSIVGLALSDLIQK--YFVSPTLFRVIRLARIGRVLRLIRGAKG
hNav1.5    KQAFDVTIMFLICLNMVTMMVETDDQSPEKINILAKINLLFVAIFTGECIVKLAALRHYYFTNSWNIFDFVVVILSIVGTVLSDIIQK--YFFSPTLFRVIRLARIGRILRLIRGAKG
hNav 1.6   QQAFDIVIMMLICLNMVTMMVETDTQSKQMENILYWINLVFVIFFTCECVLKMFALRHYYFTIGWNIFDFVVVILSIVGMFLADIIEK--YFVSPTLFRVIRLARIGRILRLIKGAKG
hNav1.7    NQAFDITIMVLICLNMVTMMVEKEGQTDYMSFVLYWINVVFIILFTGECVLKLISLRHYYFTVGWNIFDFVVVILSIVGMFLAEMIEK--YFVSPTLFRVIRLARIGRILRLIKGAKG
hNav1.8    RQAFDITIMVLICLNMITMMVETDDQSEEKTKILGKINQFFVAVFTGECVMKMFALRQYYFTNGWNVFDFIVVVLSIASLIFSAILKSLQSYFSPTLFRVIRLARIGRILRLIRAAKG
hNav 1.9   SQIFDIIIISLIILNMISMMAESYNQPKAMKSILDHLNWVFVVIFTLECLIKIFALRQYYFTNGWNLFDCVVVLLSIVSTMISTLENQEHIPFPPTLFRIVRLARIGRILRLVRAARG
```

Rattus norvegicus

Mus musculus

Homo sapiens

Figure S4. MSA of VSD (DIV) of Na⁺ channels from three mammalian species (*Rattus norvegicus*, *Mus musculus* and *Homo sapiens*).

```
gi|403447|D. melanogaster    DKKFDIIIMLFIGLNMFTMTLDRYDASDTYNAVLDYLNAIFVVIFSSECLLKIFALRYHYFIEPWNLFDVVVVILSIILGLVLSDIIEKYFVSPTLLRVVRVAKVGRVLRLVKGAKG
gi|194893768|D. erecta       DKKFDIIIMLFIGLNMFTMTLDRYDASETYNAVLDYLNAIFVVIFSSECLLKIFALRYHYFIEPWNLFDVVVVILSIILGLVLSDIIEKYFVSPTLLRVVRVAKVGRVLRLVKGAKG
gi|195132819|D. mojavensis   DKKFDIIIMLFIGLNMFTMTLDRYDASDTYNAVLDYLNAIFVVIFSSECLLKIFALRYHYFIEPWNLFDVVVVILSIILGLVLSDIIEKYFVSPTLLRVVRVAKVGRVLRLVKGAKG
gi|195048329|D. grimshawi    DKKFDIIIMLFIGLNMFTMTLDRYDASDTYNAVLDYLNAIFVVIFSSECLLKIFALRYHYFIEPWNLFDVVVVILSIILGLVLSDIIEKYFVSPTLLRVVRVAKVGRVLRLVKGAKG
gi|194768088|D. ananassae    DKKFDIIIMLFIGLNMFTMTLDRYDASDTYNAVLDYLNAIFVVIFSSECLLKIFALRYHYFIEPWNLFDVVVVILSIILGLVLSDIIEKYFVSPTLLRVVRVAKVGRVLRLVKGAKG
gi|195447454|D. willistoni   DKKFDIIIMLFIGLNMFTMTLDRYDASDTYNAVLDYLNAIFVVIFSSECLLKIFALRYHYFIEPWNLFDVVVVILSIILGLVLSDIIEKYFVSPTLLRVVRVAKVGRVLRLVKGAKG
gi|380690198|B. dorsalis     DKKFDIIIMLFIGLNMFTMTLDRYDASETYNAVLDYLNAIFVVIFSSECLLKIFALRYHYFIEPWNLFDVVVVILSIILGLVLSDIIEKYFVSPTLLRVVRVAKVGRVLRLVKGAKG
```

gi|195479236|*D. yakuba*	DKKFDIIIMLFIGLNMFTMTLLRYDASDTYNAVLDYLNAIFVVIFSSECLLKIFALRYHYFIEPWNLFDVVVVILSIIGLVLSDIIEKYFVSPTLLRVVRVAKVGRVLRLVKGAKG
gi|198468369|*D. pseudoobscura*	DKKFDIVIMLFIGLNMFTMTLLRYDASDTYNAVLDYLNAIFVVIFSSECLLKIFALRYHYFIEPWNLFDVVVVILSIIGLVLSDIIEKYFVSPTLLRVVRVAKVGRVLRLVKGAKG
gi|499007704|*C .capitata*	DKKFDIIIMLFIGLNMFTMTLLRYDASETYNAVLDYLNAIFVVIFSSECLLKIFALRYHYFIEPWNLFDVVVVILSIIGLVLSDIIEKYFVSPTLLRVVRVAKVGRVLRLVKGAKG
gi|1842214|*M. domestica*	DKKFDIIIMLFIGLNMFTMTLLRYDASEAYNNVLDKLNGIFVVIFSGECLLKIFALRYHYFKEPWNLFDVVVVILSIIGLVLSDIIEKYFVSPTLLRVVRVAKVGRVLRLVKGAKG
gi|148291097|*A .gambiae*	NKKFDMIIMLFIGFNMLTMTLLHYKQSETFSAVLDYLNMIFICIFSSECLMKIFALRYHYFIEPWNLFDFVVVILSIIGLVLSDIIEKYFVSPTLLRVVRVAKVGRVLRLVKGAKG
gi|290349620|*C. pallens*	NKKFDMIIMLFIGFNMLTMTLLHYKQSDTFSAVLDYLNMIFICIFSSECLMKIFALRYHYFIEPWNLFDFVVVILSIIGLVLSDLIEKYFVSPTLLRVVRVAKVGRVLRLVKGAKG
gi|290349618|*C. quinquefasciatus*	NKKFDMIIMLFIGFNMLTMTLLHYKQTETFSAVLDYLNMIFICIFSSECLMKIFALRYHYFIEPWNLFDFVVVILSIIGLVLSDLIEKYFVSPTLLRVVRVAKVGRVLRLVKGAKG
gi|214011860|*A. aegypti*	NKKFDMIIMLFIGFNMLTMTLLHYKQTDTFSAVLDYLNMIFICIFSSECLMKIFALRYHYFIEPWNLFDFVVVILSIIGLVLSDLIEKYFVSPTLLRVVRVAKVGRVLRLVKGAKG
gi|668455755|*A. sinensis*	NKKFDMIIMLFIGFNMLTMTLLHYKQSETFSAVLDYLNMIFICIFSSECLMKIFALRYHYFIEPWNLFDFVVVILSIIGLVLSDLIEKYFVSPTLLRVVRVAKVGRVLRLVKGAKG
gi|50080864|*A. albopictus*	NKKFDMIIMLFIGFNMLTMTLLHYKQTDTFSAVLDYLNMIFICIFSSECLMKIFALRYHYFIEPWNLFDFVVVILSIIGLVLSDLIEKYFVSPTLLRVVRVAKVGRVLRLVKGAKG
gi|571548120|*A. mellifera*	DKKFDMIIMLFIGLNMLTMTLLHYQQTQTFSDVLDYLNMIFIVIFTSECLMKIFALRYHYFKEPWNLFDFVVVILSIIGLVLSDIIEKYFVSPTLLRVVRVAKVGRVLRLVKGAKG
gi|572270136|*A. dorsata*	DKKFDMIIMLFIGLNMLTMTLLHYQQTQTFSDVLDYLNMIFIVIFTSECLMKIFALRYHYFKEPWNLFDFVVVILSIIGLVLSDIIEKYFVSPTLLRVVRVAKVGRVLRLVKGAKG
gi|148277534|*N. vitripennis*	DKKFDMIIMLFIGLNMLTMTLLHYQQSATFSNVLDYLNMIFIVIFTSECLMKIFALRYHYFKEPWNLFDFVVVILSIIGLVLSDIIEKYFVSPTLLRVVRVAKVGRVLRLVKGAKG
gi|307210096|*H. saltator*	DKKFDMIIMLFIGLNMLTMTLLHYQQSDTFSNVLDYLNMIFIVIFTSECLLKIFALRYHYFKEPWNLFDFVVVILSIIGLVLSDIIEKYFVSPTLLRVVRVAKVGRVLRLVKGAKG
gi|665817672|*M. demolitor*	DKKFDMIIMLFIGLNMLTMTLLHYQQTATFSNVLDYLNMIFIVIFSSECLMKIFALRYHYFKEPWNLFDFVVVILSIIGLVLSDIIEKYFVSPTLLRVVRVAKVGRVLRLVKGAKG
gi|383858782|*M. rotundata*	DKKFDMIIMLFIGLNMLTMTLLHYQQTQTFSDVLDYLNMIFIVIFTSECLMKIFALRYHYFKEPWNLFDFVVVILSIIGLVLSDIIEKYFVSPTLLRVVRVAKVGRVLRLVKGAKG
gi|340718624|*B. terrestris*	DKKFDMIIMLFIGLNMLTMTLLHYQQTQTFSDVLDYLNMIFIVIFTSECLMKIFALRYHYFKEPWNLFDFVVVILSIIGLVLSDIIEKYFVSPTLLRVVRVAKVGRVLRLVKGAKG
gi|307168143|*C. floridanus*	DKKFDMIIMLFIGLNMLTMTLLHYQQSETFSNVLDYLNMIFIVIFTSECLMKIFALRYHYFKEPWNLFDFVVVILSIIGLVLSDIIEKYFVSPTLLRVVRVAKVGRVLRLVKGAKG
gi|350409729|*B. impatiens*	DKKFDMIIMLFIGLNMLTMTLLHYQQTQTFSDVLDYLNMIFIVIFTSECLMKIFALRYHYFKEPWNLFDFVVVILSIIGLVLSDIIEKYFVSPTLLRVVRVAKVGRVLRLVKGAKG
gi|607353560|*C. biroi*	DKKFDMIIMLFIGLNMLTMTLLHYQQSTTFSNVLDYLNMIFIVIFTSECLMKIFALRYHYFKEPWNLFDFVVVILSIIGLVLSDIIEKYFVSPTLLRVVRVAKVGRVLRLVKGAKG
gi|380028083|*A. florea*	DKKFDMIIMLFIGLNMLTMTLLHYQQTQTFSDVLDYLNMIFIVIFTSECLMKIFALRYHYFKEPWNLFDFVVVILSIIGLVLSDIIEKYFVSPTLLRVVRVAKVGRVLRLVKGAKG
gi|217330564|*B. mori*	DKKFDMIIMLFIGLNMLTMTLLHYQQSETFSAVLDYLNMIFIVIFSSECLLKIFALRYHYFVEPWNLFDFVVVMFSIISLVLSDIIEKYFVSPTLLRVVRVAKVGRVLRLVKGAKG
gi|357623276|*D. plexippus*	DKKFDMIIMLFIGLNMLTMTLLHYQQSDTFSAVLDYLNMIFIVIFSSECLLKIFALRYHYFVEPWNLFDFVVVMFSIISLVLSDIIEKYFVSPTLLRVVRVAKVGRVLRLVKGAKG
gi|469664985|*C. medinalis*	DKKFDMIIMLFIGFNMLTMTLLHYQQSDTFSHVLDYLNMIFIVIFSSECLLKIFALRYHYFVEPWNLFDFVVVMFSIISLVLSDIIEKYFVSPTLLRVVRVAKVGRVLRLVKGAKG
gi|197927081|*P. xylostella*	DKKFDMIIMLFIGLNMLTMTLLHYQQAESFSVVLDYLNMIFIVIFSSECMSKIFALRYHYFVDPWNWFDFVVVMFSIISLVLSDIIEKYFVSPTLLRVVRVAKVGRVLRLVKGAKG
gi|189172241|*B. mandarina*	DKKFDMIIMLFIGLNMLTMTLLHYQQSETFSAVLDYLNMIFIVIFSSECLLKIFALRYHYFVEPWNLFDFVVVMFSIISLVLSDIIEKYFVSPTLLRVVRVAKVGRVLRLVKGAKG
gi|295136555|*H. zea*	DKKFDMIIMLFIGLNMLTMTLLHYQQSETFSTVLDYLNMIFIVIFSSECLLKMFALRYHYFVEPVSLFDFVVVNFSIISLVLSDIIEKYFVSPTLLRVVRVAKVGRVLRLVKGAKG
gi|3320596|*H. virescens*	DKKFDMIIMLFIGLNMLTMTLLHYQQSETFSTVLDYLNMIFIVIFSSECLLKMFALRYHYFVEPWNLFDFVVVNFSIISLVLSDIIEKYFVSPTLLRVVRVAKVGRVLRLVKGAKG
gi|500866367|*L. bostrychophila*	NTKFDMIIMLFIGLNMLTMTLLHYQQSETFSNVLDTLNLIFIVIFSSECMMKIFALRYHYFVEPWNLFDFVVVILSIIGLVLSDIIEKYFVSPTLLRVVRVAKVGRVLRLVKGAKG
gi|29467444|*P. corporis*	NTKFDMIIMLFIGLNMLTMTLLHYKQTETFSKVLDTLNLIFIVIFSSECLMKIFALRYHYFVEPWNLFDFVVVILSIIGLVLSDIIEKYFVSPTLLRVVRVAKVGRVLRLVKGAKG
gi|30350262|*P. humanus*	NTKFDMIIMLFIGLNMLTMTLLHYKQTETFSKVLDTLNLIFIVIFSSECLMKIFALRYHYFVEPWNLFDFVVVILSIIGLVLSDIIEKYFVSPTLLRVVRVAKVGRVLRLVKGAKG
gi|260528198|*P. americana*	NKKFDMIIMLFIGFNMLTMTLLHYQQSQQFSDVLDYLNMIFIVIFSSECLMKIFALRYHYFKEPWNLFDFVVVILSIIGLVLSDIIEKYFVSPTLLRVVRVAKVGRVLRLVKGAKG
gi|1657986|*B. germanica*	DKKFDMIIMLFIGFNMLTMTLLHYQQSKQFSDVLDYLNMIFIVIFSSECLMKIFALRYHYFKEPWNLFDFVVVILSIIGLVLSDIIEKYFVSPTLLRVVRVAKVGRVLRLVKGAKG
gi|259906459|*T. castaneum*	NKKFDMIIMLFIGLNMLTMTMLHYQQKETFTKVLDSLNMIFIVIFSTECLMKIFALRYHYFTEPWNLFDLVVVILSIIGLVLSDIIEKYFVSPTLLRVVRVAKVGRVLRLVKGAKG
gi|546673724|*D. ponderosae*	NKKFDMFIMLFIGLNMLTMTMLHYQQKETFTKVLDYLNMIFIVIFTTECLMKVFALRYHYFTEPWNLFDLVVVILSIIGLVLSDIIEKYFVSPTLLRVVRVAKVGRVLRLVKGAKG
gi|646703172|*Z. nevadensis*	NKKFDMIIMLFIGFNMLTMTMLHYQQSKRFSDVLDYLNMIFIVIFSSECLMKIFALRYHYFKEPWNLFDFVVVILSIIGLVLSDIIEKYFVSPTLLRVVRVAKVGRVLRLVKGAKG
gi|589908288|*T. palmi*	NKKFDMIIMLFIGFNMLTMTLLHYQQSETFSMVLDHLNMIFIVIFSSECLMKVFALRYHYFVEPWNLFDFVVVILSIIGLVLSDIIEKYFVSPTLLRVVRVAKVGRVLRLVKGAKG
gi|209361483|*C. lectularius*	DKKFDMIIMLFIGFNMLTMTMLHYQQTEMFSFVLDILNMIFIVIFSSECLLKIFALRYHYFKEPWNLFDFVVVLLSIIGLVLSDIIEKYFVSPTLLRVVRVAKVGRVLRLVKGAKG

Figure S5. MSA of VSD (DIV) of insect Na^+ channels. These sequences are derived from nine insect Orders (bar in grey: *Diptera*; purple: *Hymenoptera*; orange: *Lepidoptera*; red: *Phthiraptera*; pale green: *Blattodea*; green: *Coleoptera*; pale blue: *Thysanoptera*; blue: *Blattaria*; black: *Hemiptera*).

Supplementary Table 1 Parameter estimates and likelihood ratio statistics (2Δ*l*) for the scorpion α-toxin gene from *M. martensii*

Models	*p*	*l*	Parameter estimates	2Δ*l*	PSS
M0 (one-ratio)	1	-1497.0	$\omega = 0.85$		None
M1a (neutral)	2	-1448.0	$p_0 = 0.46$ ($p_1 = 0.54$)		Not allowed
			$\omega_0 = 0.08$ ($\omega_1 = 1$)	16.6	
M2a (selection)	4	-1439.7	$p_0 = 0.42$		2R*, 8K*, 9P*, 15E, 17A*,
			$p_1 = 0.30$ ($p_2 = 0.27$)		18R**, 37Q, 38W, 39V*, 54N
			$\omega_0 = 0.09$ ($\omega_1 = 1$)		
			ω_2 = **2.47**		
M7 (beta)	2	-1450.2	$p = 0.20$, $q = 0.15$	20.0	Not allowed
M8 (beta & ω)	4	-1440.2	$p_0 = 0.64$ ($p_1 = 0.38$)		2R*, 8K**, 9P**, <u>10H</u>, <u>13V*</u>, 15E*,
			$p = 0.32$, $q = 0.58$		17A*, 18R**, 37Q*, 38W*, 39V**,
			ω_s =**2.21**		54N*

Note: *p* is the number of parameters in the ω distribution; *l* is the log likelihood. Twice the log likelihood differences (2Δ*l*) between null models and their alternative models: M1a/M2a=16.6 and M7/M8=20.0, both having χ^2 significant values of $p < 0.001$. PSSs identified by the Naïve Empirical Bayes (NEB) methods under M2a and M8 with P (posterior probabilities) ≥ 0.9 are shown and those ≥ 0.99 are indicated by ** and ≥ 0.95 by *. Residues are numbered according to BmKM1. Two ω values in M2a and M8, as indicators of positive selection, are boldfaced. M8-specific PSSs are underlined once.

Supplementary Table 2 Log-likelihood values and parameter estimates under the fixed-sites models for Na_v channel genes of birds

Model	*p*	*l*	r_2	κ	ω
A (homogeneous)	177	-6494.05	1	2.74	0.091
B (different *r*s)	178	-6468.83	1.62	2.68	0.090
C (different *r*s and πs)	187	-6454.74	1.41	2.57	0.090
D (different *r*s , κ and ω)	180	-6459.54	1.51	$\kappa_1 = 2.50$	$\omega_1 = 0.073$
				$\kappa_2 = 3.35$	$\omega_2 = 0.136$
E (different *r*s, κ, ω and πs)	189	-6447.52	1.33	$\kappa_1 = 2.55$	$\omega_1 = 0.074$
				$\kappa_2 = 2.87$	$\omega_2 = 0.131$
F (separate analysis)	354	-6340.11	1.52	$\kappa_1 = 2.53$	$\omega_1 = 0.077$
				$\kappa_2 = 2.92$	$\omega_2 = 0.120$

Note: *p* is the number of parameters including 166 branch lengths in the tree. The first partition refers to the two extracellular loops and the second the remaining portion of the VSD. r_2 is the rate of the second site partition relative to the rate of the first partition ($r_1 = 1$).

Supplementary Table 3 Log-likelihood values and parameter estimates under the fixed-sites models for Na_v channel genes of lizards

Model	*p*	*l*	r_2	κ	ω
A (homogeneous)	65	-3720.13	1	2.19	0.070
B (different *r*s)	66	-3689.90	2.20	2.13	0.064
C (different *r*s and πs)	75	-3683.19	1.76	2.12	0.064
D (different *r*s, κ and ω)	68	-3673.95	1.45	κ_1 = 2.54	ω_1 = 0.047
				κ_2 = 1.68	ω_2 = 0.124
E (different *r*s, κ, ω and πs)	77	-3666.68	1.18	κ_1 = 2.60	ω_1 = 0.047
				κ_2 = 1.58	ω_2 = 0.119
F (separate analysis)	130	-3615.89	1.77	κ_1 = 2.53	ω_1 = 0.049
				κ_2 = 1.72	ω_2 = 0.102

Note: *p* is the number of parameters including 54 branch lengths in the tree. Meanings for the two partitions of VSD and r_2 are the same with those in Supplementary Table 2 (the same below).

Supplementary Table 4 Log-likelihood values and parameter estimates under the fixed-sites models for Na_v channel genes of mammals

Model	*p*	*l*	r_2	κ	ω
A (homogeneous)	63	-3743.57	1	2.57	0.071
B (different *r*s)	64	-3675.42	3.00	2.59	0.067
C (different *r*s and πs)	73	-3663.73	2.58	2.54	0.065
D (different *r*s , κ and ω)	66	-3655.35	2.06	κ_1 = 2.90	ω_1 = 0.043
				κ_2 = 2.37	ω_2 = 0.135
E (different *r*s, κ and πs)	75	-3642.12	1.72	κ_1 = 3.05	ω_1 = 0.042
				κ_2 = 2.15	ω_2 = 0.130
F (separate analysis)	126	-3594.72	1.62	κ_1 = 3.12	ω_1 = 0.038
				κ_2 = 2.28	ω_2 = 0.123

Note: *p* is the number of parameters including 52 branch lengths in the tree.

Supplementary Table 5 Log-likelihood values and parameter estimates under the fixed-sites models for Na_v channel genes of insects

Model	*p*	*l*	r_2	κ	ω
A (homogeneous)	99	-4777.52	1	1.48	0.015
B (different rs)	100	-4749.93	2.11	1.42	0.013
C (different rs and πs)	109	-4747.02	1.71	1.43	0.013
D (different rs , κ and ω)	102	-4741.72	1.64	κ_1 = 1.31	ω_1 = 0.010
				κ_2 = 1.87	ω_2 = 0.024
E (different rs, κ ω and πs)	111	-4740.80	1.41	κ_1 = 1.38	ω_1 = 0.010
				κ_2 = 1.65	ω_2 = 0.021
F (separate analysis)	198	-4669.57	1.09	κ_1 = 1.38	ω_1 = 0.008
				κ_2 = 1.52	ω_2 = 0.023

Note: *p* is the number of parameters including 88 branch lengths in the tree.

Supplementary References

1. Xiong, Y. M., Ling, M. H., Wang, D. C. & Chi, C. W. The cDNA and genomic DNA sequences of a mammalian neurotoxin from the scorpion *Buthus martensii karsch*. *Toxicon* **35**, 1025-1031 (1997).
2. Luo, M. J. *et al*. Purification and sequences determination of a new neural mammalian neurotoxin from the scorpion *Buthus martensii karsch*. *Toxicon* **35**, 723-731 (1997).
3. Surtiere, P. *et al*. New toxins acting on sodium channels from the scorpion *Leiurus quinquestriatus hebraeus* suggest a clue to mammalian vs insect selectivity. *Toxicon* **36**, 1141-1154 (1998).
